# Predicting change: Approximate inference under explicit representation of temporal structure in changing environments

**DOI:** 10.1371/journal.pcbi.1006707

**Published:** 2019-01-31

**Authors:** Dimitrije Marković, Andrea M. F. Reiter, Stefan J. Kiebel

**Affiliations:** Department of Psychology, Technische Universität Dresden, Dresden, Germany; Oxford University, UNITED KINGDOM

## Abstract

In our daily lives timing of our actions plays an essential role when we navigate the complex everyday environment. It is an open question though how the representations of the temporal structure of the world influence our behavior. Here we propose a probabilistic model with an explicit representation of state durations which may provide novel insights in how the brain predicts upcoming changes. We illustrate several properties of the behavioral model using a standard reversal learning design and compare its task performance to standard reinforcement learning models. Furthermore, using experimental data, we demonstrate how the model can be applied to identify participants’ beliefs about the latent temporal task structure. We found that roughly one quarter of participants seem to have learned the latent temporal structure and used it to anticipate changes, whereas the remaining participants’ behavior did not show signs of anticipatory responses, suggesting a lack of precise temporal expectations. We expect that the introduced behavioral model will allow, in future studies, for a systematic investigation of how participants learn the underlying temporal structure of task environments and how these representations shape behavior.

## Author summary

Although time perception and timed behavior are essential for our everyday experience, it is still unclear how the human brain represents the underlying temporal regularities of our dynamic environment. These regularities and their representations in the brain are important to generate well-timed behavior. When deciding on the sequence of actions to complete most of our everyday tasks like cooking, driving, or even brushing our teeth, it is essential to represent and keep track of the durations of different parts of the tasks. Here we introduce a behavioral model of decision making in environments in which a change is at least partially predictable by the time it took since the last change. We show that human participants are using such predictions in the so-called reversal learning task, which simulates abrupt but not immediately obvious changes of the environment. We find that some but not all participants harness previously experienced regularities in these changes to anticipate when the next change is going to happen. We expect that a wide range of similar questions of how humans and other animals use temporal expectations to make their decisions in a dynamic environment can be addressed using the new modelling approach.

## Introduction

Our ability to represent time and to generate complex actions and plans based on this representation are central to all aspects of our behavior. Knowing when to act, precisely, is clearly crucial for our survival [1, 2]. Thus, it is not surprising that the question of how we perceive and represent time gains an increasing interest in neurosciences [3–5]. However, in comparison to our understanding of spatial cognition, the neural basis of time perception is still poorly understood [6]. Here, we address the question of how the brain, in principle, can use knowledge about a hidden temporal structure of a task when making decisions.

Traditionally, the question how we learn the structure of the world and use these representations for decision making, has been investigated from the perspective of reinforcement learning [7, 8]. The focus of these investigations has been on how learning is driven by prediction errors [9], defined as a mismatch between expected and observed outcomes of one’s actions. More recent studies on human behavior in dynamic environments [10–13] have demonstrated that the relative precision of one’s prior beliefs and current sensory information weights prediction error signals [14–21]. These findings indicate that humans update their beliefs about the structure of the world akin to a rational (Bayesian) agent [22, 23]. This suggests that one can approximately describe human behavior using the methodology of probabilistic inference. The key advantage of the probabilistic inference framework over the standard reinforcement learning modelling approach is that one can embed the knowledge about the structure of the world and the uncertainty about that knowledge within a generative model that describes the known rules that shape the dynamics of the world.

A potential limitation of previous approaches—both probabilistic and reinforcement learning based—is that they do not take into account the underlying temporal structure of the task. Recently a series of studies have demonstrated the relevance of learned temporal associations on attention, perception, and sensory integration [24–27]. Experimental paradigms employed in this studies utilized observable temporal structure within trial. Similarly, in [28, 29] authors demonstrate that learning of the underlying temporal structure of the task is used by human participants (or animal subjects) to anticipate the moment in time at which rules are most likely to change. In contrast to the experimental design which use within trial temporal structure, here participants were exposed to a hidden temporal structure across trials.

Motivated by these findings, we propose a way to extend current probabilistic models of behavior, aimed at describing decision making in dynamic environments, to incorporate an explicit representation of the underlying temporal structure in the form of prior beliefs about state durations. In particular we will focus on the case when the state durations are not directly observable, but inferred across multiple trials using observable changes in action outcomes. The two essential components of the proposed behavioral model are (i) the update of beliefs about states and duration derived using approximate inference under hidden semi-Markov models [30, 31] and (ii) the update of beliefs about actions, that is the planning process, cast as an inference problem [32–34].

As a test bed for illustrating the key properties of the model and for demonstrating its applicability to experimental studies we adopted a probabilistic reversal learning task [35]. This task and its variants have been frequently used in human and animal studies to investigate key properties of flexible behavioral adaptation in dynamic environments [29, 36–38]. In a typical setup participants first learn to associate a particular choice with a reward, followed by a sequence of reversals of reward contingencies. The interesting question is typically how fast participants adapt their choices to these new contingencies. In the work here, we address the question, whether participants actually use the underlying temporal task structure to predict the moment of the reversal so that behavior is adapted faster as compared to the case when the reversal is unexpected.

Using the reversal learning task, we first demonstrate using simulations that we can link sub-optimal behavior in changing environments to a misrepresentation of the underlying temporal structure of the changes. Subsequently, when fitting the behavioral model to experimental data, we relate the measured behavior of each participant to model parameters that define the beliefs about the temporal structure of reversals. Strikingly, our results suggest a heterogeneous distribution of the task representation across participants where some participants correctly inferred the latent temporal structure of the task, whereas others did not. We discuss potential reasons for this finding and how the presented approach enables systematic investigations of how participants learn the underlying temporal structure of task environments and how these representations shape behavior.

## Materials and methods

In what follows we will first introduce the reversal learning task which we used here as a test bed for illustrating behavioral properties of the proposed model and its applicability to experimental data. Afterwards, we will briefly describe a classical reinforcement learning approach for modeling behavior in a reversal learning task, and later introduce the procedure for deriving the probabilistic behavioral model based on a special type of hidden semi-Markov models. Finally, we will describe in detail the analysis steps used for fitting behavioral models to data, and performing model comparison.

### Ethics statement

All participants provided written informed consent and were paid on an hourly basis. The Medical Faculty of Leipzig University approved the study.

### Reversal learning

A probabilistic reversal learning task is typically structured as follows. An agent is presented with two choices A and B where each choice is associated with a probability of receiving a reward or punishment. For example, initially choice A has high probability *p*_*H*_ and choice B low probability *p*_*L*_ of getting a reward. Importantly, after several trials the reward contingencies switch, such that choice B now corresponds to the high reward probability choice. Participants are not informed about the switch and they have to infer that a change occurred and adapt their behavior. Here we used a multiple reversals design in which reversals occur several times during the experiment. Furthermore, the reversals occur at predefined trial numbers and are independent of participant’s performance. This reversal schedule was successfully used in past studies in healthy as well as patient samples and is well suited to detect inter-individual differences in behavioral adaptation [39, 40].

**Fig 1:**
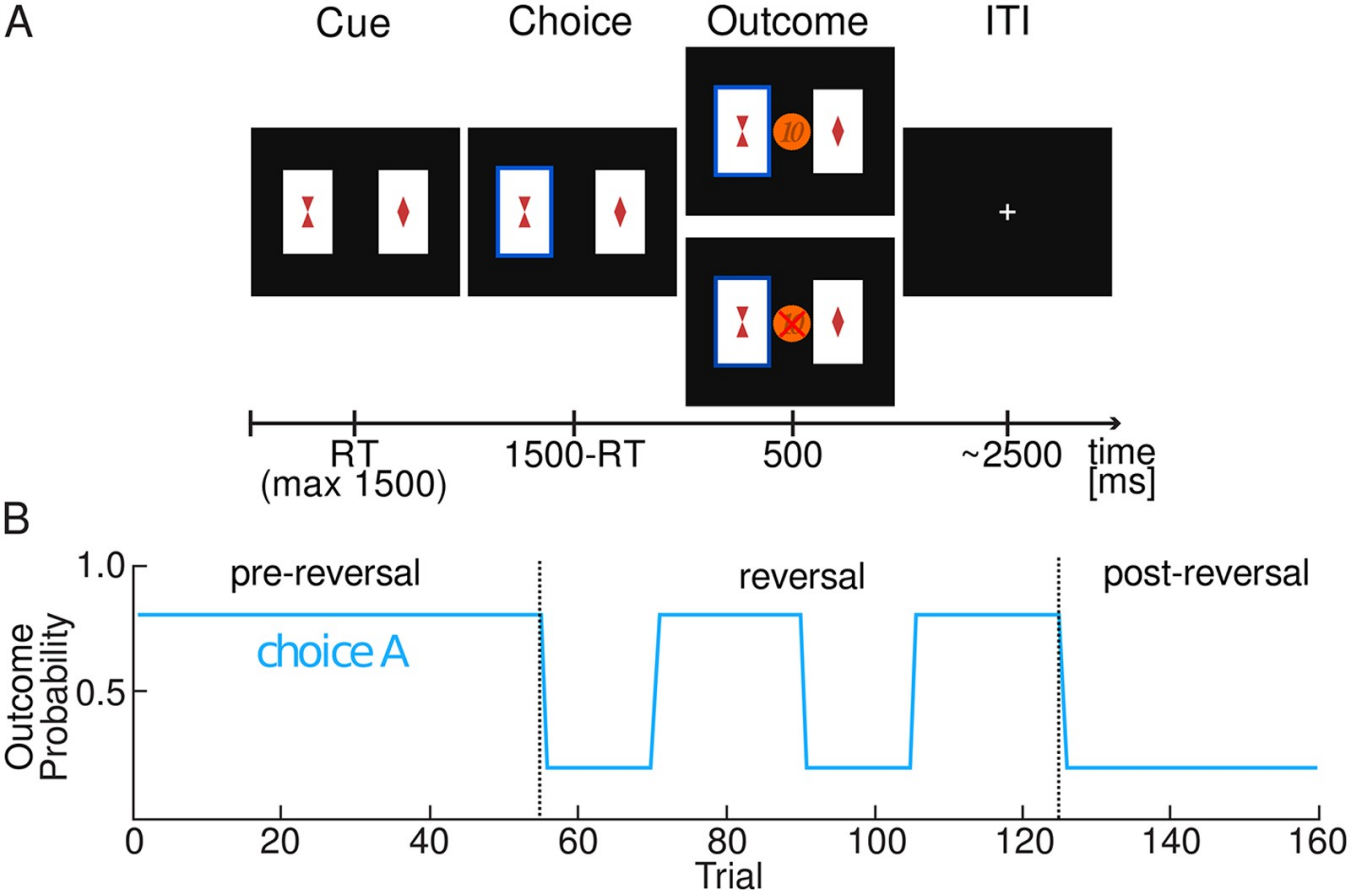
Reversal learning task. A: Exemplary trial sequence of experimental reversal learning task. Participants were instructed to choose the card that they thought would lead to a monetary reward. After they chose one of the two cards, the corresponding card was highlighted and feedback was displayed. The feedback consisted of either a 10 Eurocents coin for a win outcome, or a crossed 10 Eurocents for a loss outcome. B: The time series of the underlying reward probability of one of the two stimuli. The reward probability of the more rewarding stimuli at any time step was set to 0.8 and a punishment probability to 0.2 (and vice versa for the other stimulus). Reward contingencies remained stable for the first 55 trials (pre-reversal phase) and for the last 35 trials (post-reversal phase). In between, reward contingencies changed four times (reversal phase).

For the model-based analysis, we used a subset of behavioral data—consisting of 22 healthy participants—from a previously published fMRI study [39]. In the experimental task participants were deciding between two cards shown on a screen, each showing a different stimulus (a geometric shape, e.g. rectangle, triangle, etc.) as shown in Fig 1A. The reward probabilities associated with the two choice options were anti-correlated on all trials: whenever reward probability of choice A was high (*p*_*H*_ = 0.8) the reward probability of choice B was low (*p*_*L*_ = 0.2), and vice versa. Note that *p*_*H*_ = 1 − *p*_*L*_ on all trials. Reward contingencies change as follows: they were stable for the first 55 trials, afterwards they changed four times after 15 or 20 trials, and remained stable for the last 35 trials, see Fig 1B. In total, the experimental block consisted of 160 trials.

The location of each stimulus on the screen (right or left side) was randomized over trials. After each choice the stimulus was highlighted and depicted for 1.5 s minus the reaction time. The feedback in form of reward (won 10 Eurocents) or punishment (lost 10 Eurocents) was shown for 0.5 s. If no response occurred during the decision window, the message “too slow” was presented, and no outcome was delivered. During the inter-trial interval, a fixation cross was presented for a variable duration (jittered and exponentially distributed; range 1–12.5 s).

All 22 participants underwent a training session during which they had the opportunity to learn the statistics of the rewards associated with high and low reward probability choices. The set of stimuli used in the training phase differed from the one used during the testing phase. The participants were instructed that they could either win or lose 10 cents on each trial, and that they will be paid the total amount of money they gained during the testing phase at the end of the experiment. Each participant performed 20 training trials without any reversal of reward contingencies. Before the start of the testing phase participants were told that reward probabilities might change over the course of the experiment. No other information about reversals or the correlation of choices and outcomes was provided. Thus, the participants had no explicitly instructed knowledge about the anti-correlated task structure before the experiment.

### Reinforcement learning models

#### Classical Rescorla-Wagner model

A classical approach to modeling the probabilistic multiple-reversals learning task is using the Rescorla-Wagner (RW) model [41, 42]. The reasoning of this approach is that agents tend to value those choices which on average lead to more rewarding outcomes in the past. This reasoning can be formalized as follows

$$\begin{array}{c} V_{t+1}^{c_{t}}=V_{t}^{c_{t}}+\alpha \left(o_{t}-V_{t}^{c_{t}}\right) \end{array}$$ (1)

where *c*_*t*_ ∈ [*A*, *B*] denotes a choice at trial *t*, *o*_*t*_ ∈ {−1, 1} denotes the outcome of that choice (loss or gain), and $V_{t}^{c_{t}}$ is the current value associated with each choice. Importantly, the choice values $V_{t}^{c_{t}}$ are updated after each trial proportionally to the prediction error $\Delta_{t}=o_{t}-V_{t}^{c_{t}}$ weighted by a constant learning rate *α*.

#### Dual update Rescorla-Wagner model

As the experimental design imposes anti-correlation between high and low reward probability (*p*_*H*_ = 1 − *p*_*L*_), an extended version of Eq (1) was proposed in [39, 40] which incorporates fictive learning signals [43]. The extension is based on the assumption that agents update, in parallel, values of both choices. The value of the executed choice is updated as before, whereas the value of the alternative (non-executed) choice is updated as if the outcome for that choice was the opposite to the observed one. We can formalize this with the following update rules

$$\begin{array}{c} \begin{array}{cc} V_{t+1}^{c_{t}} & =V_{t}^{c_{t}}+\alpha \left(o_{t}-V_{t}^{c_{t}}\right)\\ V_{t+1}^{\hat{P}c_{t}} & =V_{t}^{\hat{P}c_{t}}+\kappa \alpha \left(-o_{t}-V_{t}^{\hat{P}c_{t}}\right) \end{array} \end{array}$$ (2)

where $\hat{P}$ denotes the permutation operator such that if *c*_*t*_ = *A*, then $\hat{P}c_{t}=B$ (and vice versa). In addition, *κ* denotes the coupling strength of the fictive prediction error.

### Probabilistic model

To derive the probabilistic model of behavior we start by defining a generative model of the task that formalizes an agent’s beliefs about the structure and the dynamics of the task environment. The update rules are then obtained using (approximate) Bayesian inference.

The assumption here is that participants learn to represent the latent task structure. This structure consists of hidden states which define two possible configurations of the environment: in one configuration stimulus A corresponds to a high reward choice, in the second, stimulus B corresponds to a high reward choice. Note that the notion of state used here differs from what is typically used in reinforcement learning models, in which states are cues that are associated with values over time. Here, the states are hidden and not directly observable. Furthermore, they capture a context, which defines how two stimuli (option cues) are related to actions and outcomes of those actions.

Importantly, the task environment transits from one state to another in a probabilistic manner. Here we will consider two assumptions about the dynamics of state transition probabilities: (i) the state transition probability is constant and independent of the moment of the previous change, as is the case under hidden Markov models (HMM) [44]; (ii) the state transition probability is time dependent and linked to the moment of the last change, which we will represent using hidden semi-Markov models (HSMM) [31, 45]. As the HMM corresponds to a special case of HSMM, in which state transition probabilities are constant, it is sufficient to define the behavioral model based on the HSMM assumption.

In what follows we will define the components of the generative model (observation likelihood and state dynamics) and derive the corresponding update rules.

#### Observation likelihood

The observation likelihood defines the probability of observing reward or punishment in any of the two possible states *s*_*t*_ ∈ {¬*R*, *R*} depending of the choice *c*_*t*_ ∈ {*A*, *B*} that an agent makes at a given trial *t*. Note that we have used ¬*R* to denote an initial non-reversal state (e.g., stimulus A is linked to a high reward probability), and *R* for a reversal state with switched reward contingencies. We define the observation likelihood as

$$\begin{array}{c} p\left(o_{t}|\vec{\rho },s_{t},c_{t}\right)=\{\begin{array}{cc} \rho_{c_{t}}^{(1+o_{t})/2}{\left(1-\rho_{c_{t}}\right)}^{(1-o_{t})/2}, & \text{if}\;s_{t}=\neg R,\\ \rho_{\hat{P}c_{t}}^{(1+o_{t})/2}{\left(1-\rho_{\hat{P}c_{t}}\right)}^{(1-o_{t})/2}, & \text{if}\;s_{t}=R, \end{array}, \end{array}$$ (3)

where the $\hat{P}$ again corresponds to the permutation operator (e.g. $\hat{P}A=B$). Note that the choice dependent reward probabilities (*ρ*_*A*_ and *ρ*_*B*_) are treated as random variables. Hence they are initially unknown to the agent and learned during the course of the experiment.

### State dynamics

To formalize the presence of sequential reversals, that is, transitions from one task configuration to another, we define the state transition probability as

$$\begin{array}{c} p\left(s_{t+1}|s_{t}\right)=\{\begin{array}{cc} 1-\delta , & \text{if}\;s_{t}=s_{t+1}\\ \delta , & \text{if}\;s_{t}\neq s_{t+1} \end{array} \end{array}$$ (4)

where *δ* denotes probability of transiting between distinct hidden states (e.g. from *s*_*t*−1_ = ¬*R* to *s*_*t*_ = *R*. This representation of the state transition process corresponds to a standard HMM previously used in reversal learning tasks [29, 38, 46–48].

Note that under this formulation of the state transition matrix the agent implicitly assumes that the interval (the elapsed number of trials) between two reversals follows a geometric distribution. Hence, the probability that any between reversal interval is of length *d* is given as

$$\begin{array}{c} p\left(d\right)={\left(1-\delta \right)}^{d-1}\delta ,\;\text{where}\;d\in \left\{1,2,3,\ldots \right\} \end{array}$$ (5)

with an expected dwelling time in each task configuration (mean interval length) set to $\mu =\frac{1}{\delta }$.

To derive agents with arbitrary beliefs about between reversal intervals we will use a special type of hidden semi-Markov models, the so-called explicit duration hidden Markov models (ED-HMM) [49, 50]. This will allow us to identify individual differences in the beliefs about the temporal task structure and to investigate what impact these beliefs might have on an agent’s performance.

### State durations

The ED-HMM embeds the generative model with the representation of state durations, that is, the state dwelling time *d*_*t*_ (the time spent in each state, ¬*R* or *R*). In other words, an agent will be able to form expectations about the number of trials between consecutive reversals.

Under the ED-HMM we define the state transition matrix as follows

$$\begin{array}{c} p\left(s_{t}|s_{t-1},d_{t-1}\right)=\{\begin{array}{cc} I_{2}, & \text{if}\;d_{t-1}>1,\\ J_{2}-I_{2}, & \text{if}\;d_{t-1}=1, \end{array} \end{array}$$ (6)

where *I*_2_ denotes the 2 × 2 identity matrix and *J*_2_ denotes the 2 × 2 all-ones matrix. The above relations describe a simple deterministic process for which the current state *s*_*t*_ remains unchanged as long as *d*_*t*−1_ > 1 and switches to alternative state (e.g. if *s*_*t*−1_ = *A* then *s*_*t*_ = *B*) with probability one when *d*_*t*−1_ = 1.

The transitions between state durations follow a deterministic countdown (*d*_*t*_ = *d*_*t*−1_ − 1) as long as *d*_*t*−1_ > 1; once *d*_*t*−1_ = 1 the subsequent value *d*_*t*_ is sampled from the prior over state durations *p*_0_(*d*_*t*_), that is, from the beliefs over between reversal interval. We can write this formally in the form of the transition probability as

$$\begin{array}{c} p\left(d_{t}|d_{t-1}\right)=\{\begin{array}{cc} \delta_{d_{t},d_{t-1}-1}, & \text{if}\;d_{t-1}>1,\\ p_{0}(d_{t}), & \text{if}\;d_{t-1}=1. \end{array} \end{array}$$ (7)

**Fig 2:**
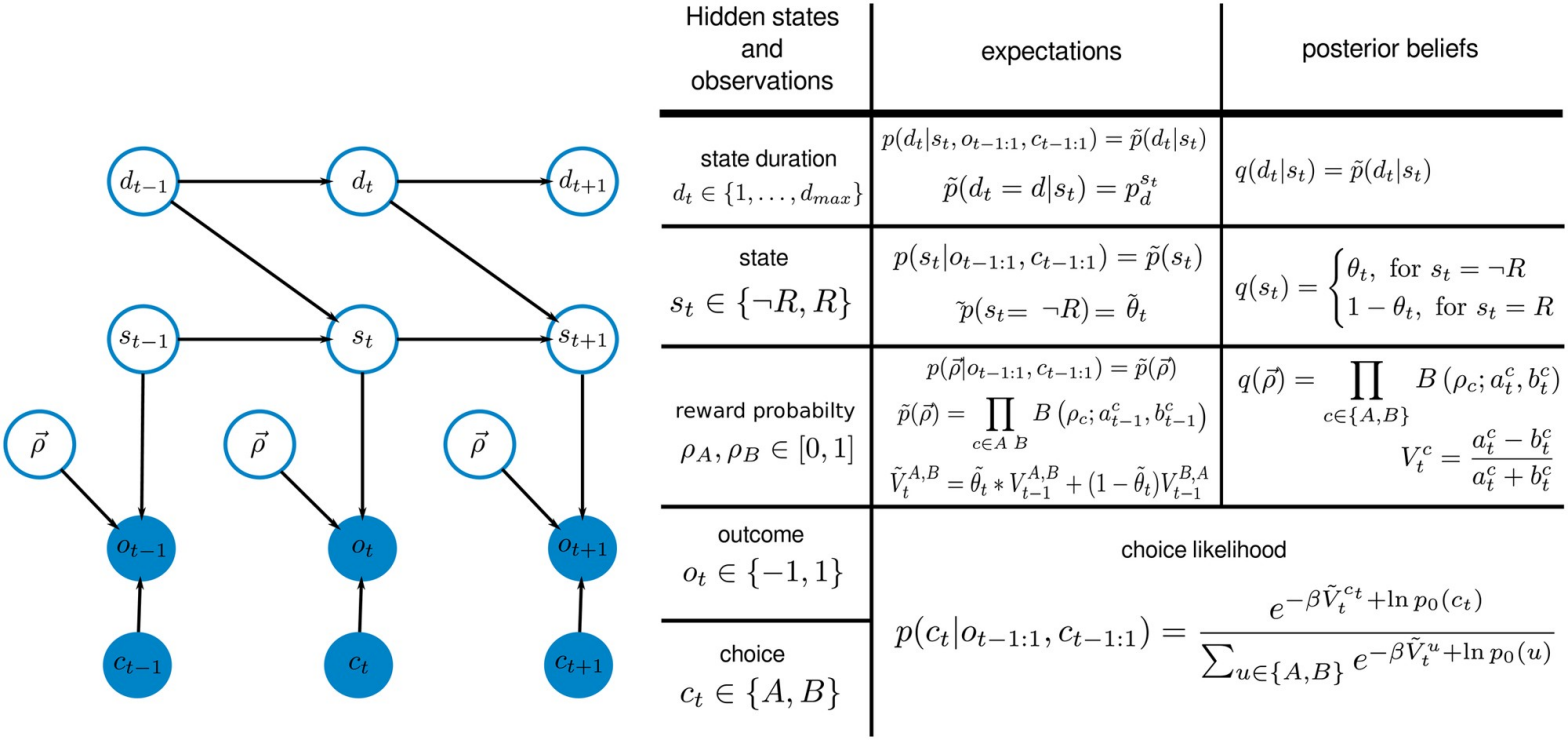
Graphical representation of the generative model and model summary. Left: We illustrate here the conditional dependence of state durations, states, reward probabilities, and outcomes. Note that the choices *c*_*t*_ are treated as observables within the generative model. Right: summary of the hidden state variables, observations, and the notation used for defining prior expectations and posterior beliefs over hidden states.

In Fig 2 we illustrate conditional dependencies between states, durations, reward probabilities, and outcomes in the form of a a graphical model.

Although, the semi-Markov formalisms allows for defining state dependent prior beliefs *p*_0_(*d*_*t*_|*s*_*t*_), here, to reduce model complexity, we have assumed that the priors are independent of the current state, thus we set *p*_0_(*d*_*t*_|*s*_*t*_) = *p*_0_(*d*_*t*_) for any *s*_*t*_ ∈ {¬*R*, *R*}.

In practice, prior beliefs about state durations *p*_0_(*d*_*t*_) can have an arbitrary form, however for the purpose of inferring the participants’ representation of between reversal intervals we will use a parametric distribution, specifically the negative binomial *NB* distribution defined as

p0(dt)=NB(dt;δ,r)=(dt+r-2dt-1)(1-δ)dt-1δr, (8)

where *δ* ∈ [0, 1] and *r* > 0.

**Fig 3:**
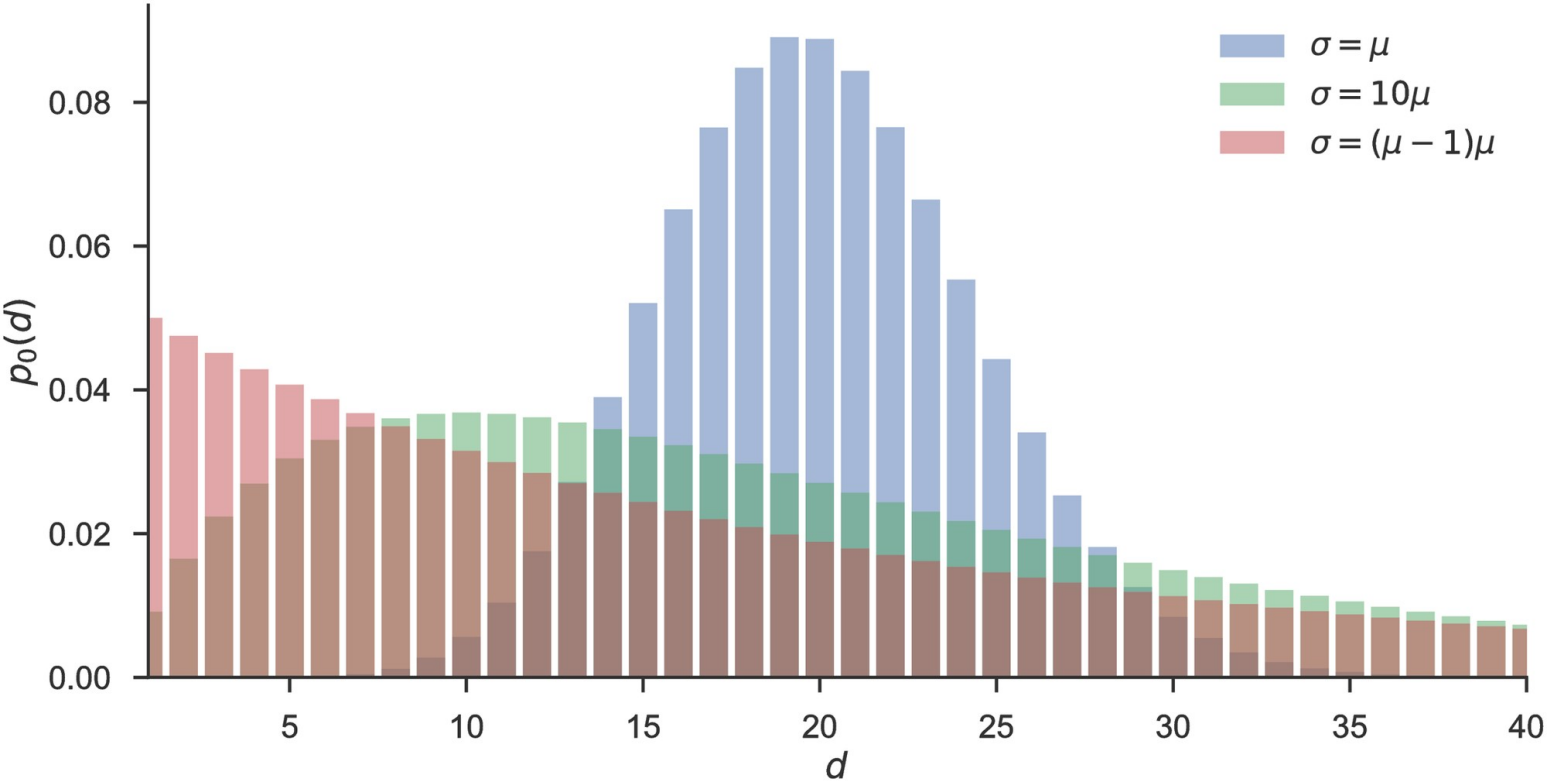
Specific cases of the negative binomial distribution. We illustrate here the dependence of the shape (and the mode) of the negative binomial distribution for different values of the variance *σ*. Note that when the variance *σ* equals its mean *μ* (blue), the distribution peaks around its expected value. As the variance increases (green) the mode shifts toward zero. The limiting case of the geometric distribution (red) corresponds to *r* = 1, that is, *σ* = *μ*(*μ* − 1). For all three cases we fixed the mean interval length at *μ* = 20.

The *NB* distribution has several convenient properties. First, in the case *r* = 1 we obtain the geometric distribution (see Eq (5)), hence we recover the HMM model described above. Second, for *r* > 1 the NB distribution exhibits a nonzero mode which shifts towards the expected value $\mu =\frac{r+\delta (1-r)}{\delta }$ as a function of a decreasing variance $\sigma =\frac{(1-\delta )r}{\delta^{2}}$, which is illustrated in Fig 3. Hence, the parameters of the negative binomial distribution will allow us to quantify an agent’s specific belief in the regularity of reversals. The lower the variance *σ*, the more an agent believes that reversals occur at regular intervals.

**Fig 4:**
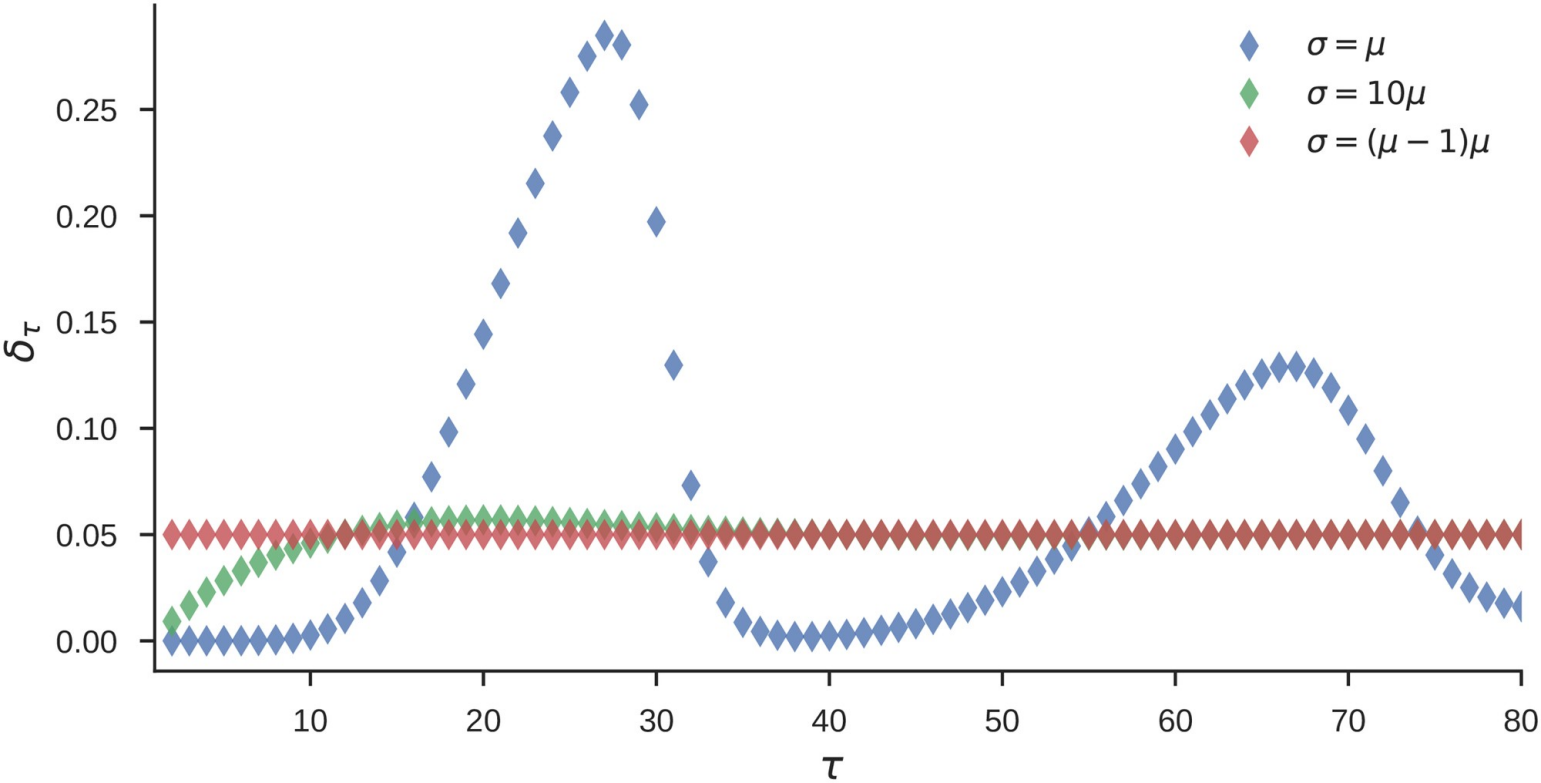
Expected transition probability at future trial *τ*. Estimate of the transition probability *δ*_*τ*_ (see Eq (9)) at a future trial *τ* conditioned upon a reversal at *τ* = 1 and known initial state set at *s*_1_ = ¬*R*. Each curve corresponds to estimates of the transition probability obtained from prior beliefs *p*_0_(*d*) shown in Fig 3. Note that for the low variance case (blue, *σ* = *μ*), the temporal profile of transition probability has clearly defined periods of low and high transition probability. As the variance increases (green) the variability of the temporal profile decreases, until it finally becomes constant in the case of the geometrically distributed prior beliefs (red, *σ* = *μ*(*μ* − 1)). For all three cases we fixed the mean interval length at *μ* = 20.

Importantly, the variance of prior beliefs *p*_0_(*d*) has direct effects on the temporal expectation profile (that a reversal will occur at some future trial *τ*). In Fig 4 we show the dependence of the expected reversal probability *δ*_*τ*_ on the variance *σ*, and under fixed mean *μ* = 20 of prior beliefs about the between-reversals interval. The expected reversal probability *δ*_*τ*_ is defined as

$$\begin{array}{c} \begin{array}{cc} \delta_{\tau } & =p(s_{\tau }=R|s_{\tau -1}=\neg R)\\ & =\sum_{d,s}p\left(s_{\tau }=R|s_{\tau -1}=\neg R,s_{1}=s,d_{1}=d\right)p_{0}(d)p(s), \end{array} \end{array}$$ (9)

where *τ* > 1, and *p*(*s* = ¬*R*) = 1. Hence, *δ*_*τ*_ corresponds to the transition probability (from non-reversal state ¬*R* into reversal state *R*) at some future trial *τ* starting from the non-reversal state *s*_1_ = ¬*R*. Note that for prior beliefs *p*_0_(*d*) with large variance (*σ* = *μ*(*μ* − 1)), we get a constant transition probability, which corresponds to the HMM formulation of the state transition probability. In contrast, for prior beliefs with low variance (*σ* = *μ*) one obtains a trial dependent transition probability with values alternating between the low and high probabilities in a periodic manner. This temporal dependence of the transition probability will affect the inference process. The agent will become insensitive to subsequent reversals occurring few trials after the initial reversal, and highly sensitive to reversals occurring twenty to thirty trials after the initial reversal.

### Inference

For model inversion we use a variational inference scheme [51–53] which allows us to derive the update rules for the inference process akin to the ones presented in Eq (2).

After making a choice *c*_*t*_ and observing outcome *o*_*t*_ at trial *t*, the agent updates its beliefs over reward probabilities $\vec{\rho }$, states *s*_*t*_, and durations *d*_*t*_ following Bayes’ rule

$$\begin{array}{c} \bar{p}\left(\vec{\rho },s_{t},d_{t}\right)\propto p\left(o_{t}|\vec{\rho },s_{t},c_{t}\right)\tilde{p}(\vec{\rho })\tilde{p}\left(d_{t}|s_{t}\right)\tilde{p}(s_{t}) \end{array}$$ (10)

where we used the following notation $\tilde{p}\left(x\right)=p\left(x|o_{t-1:1},c_{t-1:1}\right)$, and $\bar{p}\left(x\right)=p\left(x\right|o_{t:1},c_{t:1})$—for $x\in \left\{\vec{\rho },s_{t},d_{t}\right\}$—to denote prior and posterior beliefs, respectively, at time step *t*.

If we express prior beliefs as

$$\begin{array}{c} \begin{array}{cc} \tilde{p}\left(s_{t}=\neg R\right) & ={\tilde{\theta }}_{t},\\ \tilde{p}\left(d_{t}=d|s_{t}\right) & =p_{d}^{s_{t}},\\ \tilde{p}(\vec{\rho }) & =\prod_{c\in \{A,B\}}B(\rho_{c};a_{t-1}^{c},b_{t-1}^{c}), \end{array} \end{array}$$ (11)

where *B*(*x*; *a*, *b*) denotes a beta distribution, we can define the approximate posterior in similar form

$$\begin{array}{c} \bar{p}\left(\vec{\rho },s_{t},d_{t}\right)\approx q(\vec{\rho })q\left(d_{t}|s_{t}\right)q(s_{t}), \end{array}$$ (12)

where we use the following parametrization of the factors of the approximate posterior

$$\begin{array}{c} \begin{array}{cc} q(s_{t}=\neg R) & =\theta_{t},\\ q(d_{t}=d|s_{t}) & ={\bar{p}}_{d}^{s_{t}},\\ q(\vec{\rho }) & =\prod_{c\in \left\{A,B\right\}}B(\rho_{c};a_{t}^{c},b_{t}^{c}). \end{array} \end{array}$$ (13)

The prior expectation at the next trial *t* + 1 depends on the current posterior *q*(*s*_*t*_, *d*_*t*_) via the sum product rule

$$\begin{array}{c} \tilde{p}\left(s_{t+1},d_{t+1}\right)=\sum_{d_{t},s_{t}}p\left(s_{t+1},d_{t+1}|s_{t},d_{t}\right)q\left(s_{t},d_{t}\right). \end{array}$$ (14)

The update of the agent’s beliefs about reward contingencies for each choice *ρ*, current state *s*_*t*_, and the number of trials until the next reversal *d*_*t*_ is completely defined by the update rules of the sufficient statistics of the posterior, namely parameters *θ*_*t*_, $a_{t}^{c_{t}}$, and $b_{t}^{c_{t}}$. We will obtain the update rules for these parameters using variational inference. The parameters of the approximate posterior can be found as minimizers of the variational free energy defined as

$$\begin{array}{c} \begin{array}{cc} F[q] & =D_{KL}\left(q||\bar{p}\right)-\text{ln}\;\bar{p}(o_{t})\\ & =\sum_{s_{t},d_{t}}\int \text{d}\vec{\rho }q(\vec{\rho })q\left(s_{t},d_{t}\right)\text{ln}\;\frac{q(\vec{\rho })q(s_{t},d_{t})}{p\left(o_{t}|\vec{\rho },s_{t},c_{t}\right)\tilde{p}\left(\vec{\rho },s_{t},d_{t}\right)}. \end{array} \end{array}$$ (15)

The posterior beliefs *q* that minimize the free energy *F* can be obtained using the following relations

$$\begin{array}{c} q\left(\vec{\rho }\right)\propto \tilde{p}\left(\rho \right)e^{{\langle \text{ln}\;p\left(o_{t}|\vec{\rho },s_{t},c_{t}\right)\rangle }_{q\left(s_{t}\right)}},\\ q\left(s_{t},d_{t}\right)\propto \tilde{p}\left(s_{t},d_{t}\right)e^{{\langle \text{ln}\;p\left(o_{t}|{\vec{\rho }}_{t},s_{t},c_{t}\right)\rangle }_{q\left({\vec{\rho }}_{t}\right)}}. \end{array}$$ (16)

To obtain update rules similar to the delta learning rules of the RW model we simplified the above iterative procedure needed to estimate the posterior parameters. To break the cyclic update we will assume that one can first update beliefs about *q*(*s*_*t*_, *d*_*t*_) by setting

$${\langle \text{ln}\;p\left(o_{t}|\vec{\rho },s_{t},c_{t}\right)\rangle }_{q\left(\vec{\rho }\right)}\approx \text{ln}\;\tilde{p}\left(o_{t}|s_{t},c_{t}\right)$$

and then use the obtained posterior beliefs about states and durations to estimate the reward probabilities $q(\vec{\rho })$.

Using this simplification we obtain the following update rules

$$\begin{array}{ll} {\bar{p}}_{d}^{s_{t}} & =p_{d}^{s_{t}},\\ \theta_{t} & =\frac{{\tilde{\theta }}_{t}}{{\tilde{\theta }}_{t}+e^{\text{ln}\;\frac{p\left(o_{t}|s_{t}=R,c_{t}\right)}{p\left(o_{t}|s_{t}=\neg R,c_{t}\right)}}\left(1-{\tilde{\theta }}_{t}\right)}, \end{array}$$ (17)

where ${\tilde{\theta }}_{t}=\left(1-\theta_{t-1}\right)p_{d=0}^{R}+\theta_{t-1}\left(1-p_{d=0}^{\neg R}\right)$. Note that the conditional posterior *q*(*d*_*t*_|*s*_*t*_) corresponds to the conditional prior $\tilde{p}\left(d_{t}|s_{t}\right)$, as ${\bar{p}}_{d}^{s_{t}}=p_{d}^{s_{t}}$, hence it remains constant during the update of beliefs. However, the prior expectations (at the next time step) about state duration will be linked to the inference process via the state-duration transition matrix (see Eqs (6) and (7)).

Subsequently, we update the beliefs about the choice reward contingencies as

$$\begin{array}{c} \begin{array}{cc} a_{t}^{c_{t}} & =a_{t-1}^{c_{t}}+\theta_{t}{\bar{o}}_{t},\\ b_{t}^{c_{t}} & =b_{t-1}^{c_{t}}+\theta_{t}\left(1-{\bar{o}}_{t}\right),\\ a_{t}^{\hat{P}\cdot c_{t}} & =a_{t-1}^{P\cdot c_{t}}+\left(1-\theta_{t}\right){\bar{o}}_{t},\\ b_{t}^{\hat{P}\cdot c_{t}} & =b_{t-1}^{P\cdot c_{t}}+\left(1-\theta_{t}\right)\left(1-{\bar{o}}_{t}\right), \end{array} \end{array}$$ (18)

where ${\bar{o}}_{t}=\frac{o_{t}+1}{2}$ (${\bar{o}}_{t}\in \left\{0,1\right\}$).

To demonstrate the relation of the above update rules to the ones of the DU-RW model, we transform the shape parameters ($a_{t}^{c_{t}}$, and $b_{t}^{c_{t}}$) of the posterior beta distribution into the mean $\mu_{t}^{c_{t}}$ and the samples size $\nu_{t}^{c_{t}}$ as

$$\begin{array}{c} \begin{array}{cc} \nu_{t}^{x} & =a_{t}^{x}+b_{t}^{x},\\ \mu_{t}^{x} & =\frac{a_{t}^{x}}{\nu_{t}^{x}}, \end{array} \end{array}$$ (19)

where $x\in \left\{c_{t},\hat{P}\cdot c_{t}\right\}$. Expressing the expected reward probability *μ*_*t*_ as a function of the expected value *V*_*t*_, that is, as $\mu_{t}^{x}=\frac{V_{t}^{x}+1}{2}$, we get the following set of update equations

$$\begin{array}{c} \begin{array}{cc} V_{t}^{c_{t}} & =V_{t-1}^{c_{t}}+\alpha_{t}^{c_{t}}(o_{t}-V_{t-1}^{c_{t}})\\ V_{t}^{\hat{P}\cdot c_{t}} & =V_{t-1}^{\hat{P}\cdot c_{t}}+\kappa_{t}\alpha_{t}^{\hat{P}\cdot c_{t}}(o_{t}-V_{t}^{\hat{P}\cdot c_{t}}) \end{array} \end{array}$$ (20)

where $\alpha_{t}^{x}=\frac{\theta_{t}}{\nu_{t}^{x}}$, and $\kappa_{t}=\frac{1-\theta_{t}}{\theta_{t}}$.

Although in form similar to the DU-RW learning rules, a notable difference is that the fictive prediction error is of the same sign as the actual prediction error. The reason for this is that under the generative model the fictive learning signals are a product of the agent’s uncertainty about the current state of the world, that is, the current configuration of reward contingencies and not the uncertainty about the anti-correlation of reward contingencies on different choices. This alternative representation could be introduced into the generative model by introducing beliefs about a dependence between high and low reward probabilities. However, we will leave this extension for future work as it increases model complexity and does not contribute to our analysis of how temporal representations influence behavior.

### Responses

Here we will describe the response model, which defines the mapping from the agent’s beliefs to responses, based on the active inference framework [54]. We will demonstrate how a response likelihood that is often used in reinforcement learning models (in the form of a softmax transform) can be derived within this framework. We do this for didactic purposes to show how one can formally relate active inference to a well-known reinforcement learning account.

The core concept of active inference is that agents generate behavior that is most likely to minimize the expected free energy, that is, that tends to maximise, at the same time, the extrinsic and the epistemic value of agents’ choices [55]. The expected free energy can be defined as

$$\begin{array}{c} G_{t}^{c}=E_{q\left(o_{t}|c\right)}[-U(o_{t})-D_{KL}\left(q(x|o_{t},c)\left|\right|\tilde{p}(x\right))], \end{array}$$ (21)

where $x=(\vec{\rho },s_{t},d_{t})$, *U*(*o*_*t*_) denotes the utility of future outcomes (reward or punishment), and *D*_*KL*_ denotes the Kullback-Leibler divergence between the posterior (conditioned on possible future outcome and action) and prior expectations at the time step *t*. Note that we have assumed that the behavior is characterized by planning only a single time step into the future. Importantly, the response model maintains the causal structure of the task, where at trial *t* an agent first makes a choice *c*_*t*_ and only afterward observes an outcome *o*_*t*_.

The first term on the right hand side of Eq (21) is typically denoted as the extrinsic value of an action (policy) whereas the second term is denoted as epistemic value or information gain. If we express the utility *U* as

$$U\left(o_{t}\right)=\lambda o_{t},$$

where *o*_*t*_ ∈ {−1, 1}, and λ > 0, the extrinsic value becomes

$$\begin{array}{ll} {\langle U\rangle }_{q} & =\lambda {\tilde{V}}_{t}^{c},\\ {\tilde{V}}_{t}^{c} & ={\tilde{\theta }}_{t}V_{t-1}^{c}+\left(1-{\tilde{\theta }}_{t}\right)V_{t-1}^{\hat{P}\cdot c}. \end{array}$$

In practice, for sufficiently large λ the behavior will be fully driven by the extrinsic value, as each independent observation *o*_*t*_ carries little information about the underlying hidden states (a sequence of observations is required to identify the precise past moment of a reversal). In other words, each individual choice leads to a small information gain. Thus, we will assume that the expected free energy can be approximated as

$$\begin{array}{c} G_{t}^{c}\approx -\lambda {\tilde{V}}_{t}^{c}. \end{array}$$

The optimal action (choice) corresponds to the one that minimizes the expected free energy, thus

$$c_{t}=\underset{c}{\mathrm{argmin}}G_{t}^{c}=\underset{c}{\mathrm{argmax}}{\tilde{V}}_{t}^{c}.$$

Still, for describing participants’ behavior we have to assume that the action selection process is corrupted by external sources of noise; e.g. mental processes irrelevant for the task at hand. Therefore, we will soften the requirement of minimizing the expected free energy using the softmax transform, which is typically used to define the response likelihood [56, 57]. The choice probability then becomes

$$\begin{array}{c} p\left(c_{t}=c|o_{t-1:1}\right)=\frac{e^{-\beta {\tilde{V}}_{t}^{c}+\text{ln}\;p_{0}\left(c\right)}}{\sum_{u}e^{-\beta {\tilde{V}}_{t}^{u}+\text{ln}\;p_{0}\left(u\right)}}, \end{array}$$ (22)

where *β* denotes the response precision and *p*_0_(*c*) the response bias. These two parameters are the free parameters of the response model and allow us to capture participants’ deviation from optimal behavior when fitting models to the data.

Finally, in the case of the DU-RW model, we will use the same form of the response likelihood as above, with the difference that the choice value ${\tilde{V}}_{t}^{c}=V_{t}^{c}$.

### Model fitting and model comparison

When fitting models to behavior we have focused our analysis on either the DU-RW or the ED-HMM model, where we have used in both cases a hierarchical prior over free model parameters (see below for details). We will not consider the SU-RW and HMM models in model comparison as they represent special cases of the DU-RW and the ED-HMM model, respectively, which are obtained in the limit of *κ* → 0, in the case of the DU-RW model, and *r* → 1 in the case of the ED-HMM model. Hence, we can easily recover these special cases from the estimates of the posterior distributions of the free model parameters, which are summarized in Table 1.

**Table 1:** Free parameters, their transform, and interpretation for the two behavioral models. The model parameters are grouped based on whether they shape the inference (learning) or the behavioral responses. Note that the DU-RW and ED-HMM share the same mapping from beliefs into responses (see Eq (22)).

Model	Parameter	Transform	Interpretation
DU-RW	*α*	$\frac{\lambda_{1}}{1+\lambda_{1}}$	learning rate
	*κ*	$\frac{\lambda_{2}}{1+\lambda_{2}}$	coupling strength
	$V_{0}^{A}$, $V_{0}^{B}$	$\frac{\lambda_{3}-1}{\lambda_{3}+1}$, $\frac{1-\lambda_{4}}{1+\lambda_{4}}$	initial choice values
ED-HMM	*δ*	$\frac{\lambda_{1}}{1+\lambda_{1}}$	rate of prior beliefs
	*r*	1 + λ_2_	shape of prior beliefs
	$\mu_{0}^{A}$, $\mu_{0}^{B}$	$\frac{\lambda_{3}}{1+\lambda_{3}}$,$\frac{1}{1+\lambda_{4}}$	initial expectations
response	*β*	$\frac{\lambda_{5}}{1+\lambda_{5}}$	response precision
	*p*_0_(*c* = *A*)	$\frac{\lambda_{6}}{1+\lambda_{6}}$	response bias

Note that the parameters $\nu_{0}^{A}$ and $\nu_{0}^{B}$ of the ED-HMM model (see Eq (19)), which define the initial number of observations, have been fixed to values which reflect that participants underwent a 20 trials long training session. Hence, setting $\nu_{0}^{A}=\nu_{0}^{B}=10$ reflects our assumption that participants selected both options in equal amounts during the training session.

To estimate the posterior distribution over free model parameters we have used the above defined response model as observation likelihood of the behavioral model. Hence, the likelihood of behavior (the sequence of a participant’s responses) is defined as

$$\begin{array}{c} p\left(c_{T:1}^{n}|o_{T:1}^{n},\Lambda ,m\right)=\prod_{t=1}^{T}p\left(c_{t}^{n}|o_{t-1:1}^{n},\Lambda ,m\right), \end{array}$$ (23)

where $c_{T:1}^{n}$ denotes the sequence of responses of the *n*th participant (*n* ∈ {1, …, 22}), $o_{T:1}^{n}$ denotes the sequence of observations (wins or losses) that the *n*th participant made, Λ denotes the set of free model parameters, and *m* ∈ {1, 2} denotes the corresponding behavioral model.

To define the prior distribution over model parameters we have used the so-called horseshoe prior as a weakly informative hierarchical prior. If we denote the *i*th model parameter (where *i* ∈ {1, …, 6}) of the *n*th participant as $\lambda_{i}^{n}$, the horseshoe prior is defined as

$$\begin{array}{c} p\left(\lambda_{i}^{n},\tau_{i}\right)=C^{+}\left(\lambda_{i}^{n};0,\tau_{i}\right)C^{+}\left(\tau_{i};0,1\right) \end{array}$$ (24)

where *C*^+^(*x*; 0, *γ*) denotes a half-Cauchy distribution with scale parameter *γ*. Hence, the full hierarchical prior for the model *m* can be expressed as

$$\begin{array}{c} p\left(\lambda ,\tau |m\right)=\prod_{n=1}^{22}\prod_{i=1}^{6}C^{+}\left(\lambda_{i}^{n},0,\tau_{i}\right)C^{+}\left(\tau_{i};0,1\right). \end{array}$$ (25)

Note that *τ*_*i*_ acts as a hyper-prior, which plays the role of regulating the prior scale, for the corresponding free parameter, over the whole group. As $\lambda_{i}^{n}$ is a positive definite variable, we have used specific transforms to relate each $\lambda_{i}^{n}$ parameter to the corresponding model parameter (see Table 1).

The posterior probability over model parameters can be obtain from the Bayes rule as follows

$$\begin{array}{c} p\left(\lambda ,\tau |C,O,m\right)\propto \prod_{n=1}^{22}p\left(c_{T:1}^{n}|o_{T:1}^{n},\lambda ,m\right)p\left(\lambda ,\tau |m\right), \end{array}$$ (26)

where $C=\left\{c_{T:1}^{1},\ldots ,c_{T:1}^{22}\right\}$, and $O=\left\{o_{T:1}^{1},\ldots ,o_{T:1}^{22}\right\}$. However, the exact posterior distribution of the model parameters and their hyper-priors is analytically intractable, hence we have applied the variational mean-field approximation, in which one assumes that the posterior is fully factorized

$$\begin{array}{c} p\left(\lambda ,\tau |C,O,m\right)\approx \prod_{i}\prod_{n}q(\lambda_{i}^{n}\left|m\right)q(\tau_{i}\left|m\right). \end{array}$$ (27)

The full hierarchical model was implemented in the probabilistic programing library PyMC3 [58]. The PyMC3 library provides an interface to multiple state-of-the-art inference schemes. In our specific case, for estimating the approximate posterior distribution over model parameters, we have used the PyMC3 implementation of the automatic differentiation variational inference (ADVI) [59]. ADVI is a stochastic black-box variational inference scheme which returns an approximate estimate of the posterior distribution in a fully factorized form.

To attribute participants’ behavior to a specific behavioral model we have assumed that models can be treated as random effects (variables), that is, the attribution of a model to participants’ behavior can differ across participants [60]. Furthremore, we have based Bayesian model comparison on the posterior predictive model evidence [61] instead of the full model evidence, that is marginal likelihood. The motivation for basing model comparison on the posterior predictive model evidence comes from the fact that the posterior predictive model evidence is less sensitive to a prior specification of free model parameters: the approximate estimate of the predictive evidence is based on the samples from the posterior distribution which is already constrained by the subset of the behavioral data. This procedure provides for a more robust model comparison and testing results as it resolves some issues arising when using weakly-informative or misspecified prior probabilities [61]. The posterior predictive model evidence is defined as a marginal expectation of the subset of behavioral responses *C*_*T*:*k*_ condition on the behavioral responses up to the *k*th trial (*C*_*k*:1_) and the full set of outcomes presented to participants (*O*). Formally, we can define the posterior predictive model evidence as

$$\begin{array}{c} \begin{array}{cc} p(C_{T:k}|C_{k:1},O,m) & =\prod_{n}p\left(c_{T:k}^{n}|C_{k:1},O,m\right)\\ p\left(c_{T:k}^{n}|C_{k:1},O,m\right) & =\int \text{d}\lambda \prod_{t=k}^{T}p\left(c_{t}^{n}|o_{t-1:1}^{n},\lambda^{n},m\right)q\left(\lambda^{n}|m\right)\\ & \approx \frac{1}{N}\sum_{l=1}^{N}\prod_{t=k}^{T}p\left(c_{t}^{n}|o_{t-1:1}^{n},\lambda_{l}^{n},m\right) \end{array} \end{array}$$ (28)

where $q\left(\lambda^{n}|m\right)=\prod_{i}q(\lambda_{i}^{n}\left|m\right)$, and *N* = 10^4^. Note that to estimate the posterior predictive model evidence we have first estimated the approximate posterior over free model parameters on a reduced data set consisting of the pre-reversal and reversal phases (*k* = 125). Then, we generated *N* samples from the posterior distribution to estimate the posterior predictive model evidence on the remaining part of behavioral data *C*_*T*:*k*_ which covers only the post-reversal phase (see Fig 1).

We then used the posterior predictive model evidence as the likelihood of the random effect model proposed in [60]. The goal here is to identify the posterior probability that each of the two behavioral models can generate the same sequence of behavioral response as participants. Here we have defined the random effect model as the following mixture model

$$\begin{array}{c} p\left(C_{T:k},\pi ,\gamma |C_{k:1},\hat{O}\right)=p\left(C_{T:k}|C_{k:1},\hat{O},\pi \right)p\left(\pi |\gamma \right)p\left(\gamma \right), \end{array}$$ (29)

where

$$\begin{array}{c} \begin{array}{cc} p\left(C_{T:k}|C_{k:1},\hat{O},\pi \right) & =\prod_{n=1}^{22}\sum_{m_{n}=1}^{2}p\left(c_{T:k}^{n}|C_{k:1},O,m_{n}\right)p\left(m_{n}|\pi \right)\\ p(m_{n}|\pi ) & =\prod_{l=1}^{2}\pi_{l}^{\delta_{m_{n},l}},\\ p\left(\pi |\gamma \right) & =\frac{1}{B\left(\frac{1}{\gamma },\frac{1}{\gamma }\right)}\prod_{l=1}^{2}\pi_{l}^{\frac{1}{\gamma }-1}\\ p(\gamma ) & =C^{+}\left(\gamma ;0,1\right) \end{array} \end{array}$$ (30)

Similar to the role of the hyperpriors on the parameters of the behavioral model, *γ* plays here the role of a regularization parameter that allows for data driven constraints of the random effects assumption. If the marginal posterior probability over *γ* shrinks towards zero, this implies that data supports a null hypothesis which states that all models are present in the population with the same frequency and any differences we observe are purely chance driven [60].

As above, the random effects model selection was implemented in PyMC3, where the estimation of the posterior was performed using the PyMC3 implementation of the ADVI procedure. A fully factorized approximate posterior

$$\begin{array}{c} p\left(\pi ,\gamma |C,O\right)\approx q\left(\gamma \right)q\left(\pi \right) \end{array}$$ (31)

provides us with an estimate of the posterior model probability *q*(*π*). The posterior model probability can then be used to identify group level posterior estimate over possible model frequencies in the group of participants and the participant specific posterior model probability.

## Results

### Simulating behavior

To illustrate how the agent’s performance depends on the agent’s prior beliefs over the between reversal interval *p*_0_(*d*) we will start by comparing two special cases of the ED-HMM based behavioral model using synthetic data. In the first case, we will fix the variance *σ* of the prior beliefs *p*_0_(*d*) to *σ* = *μ*(1 − *μ*), which we named irregular reversal interval (IRI) agent. In the second case, we will fix the variance to *σ* = *μ*, which we named regular reversal interval (RRI) agent. In addition, we will compare the performance of the two probabilistic behavioral models to the single (SU) and dual update (DU) Rescorla-Wagner (RW) models introduced in Eqs (1) and (2), respectively.

**Fig 5:**
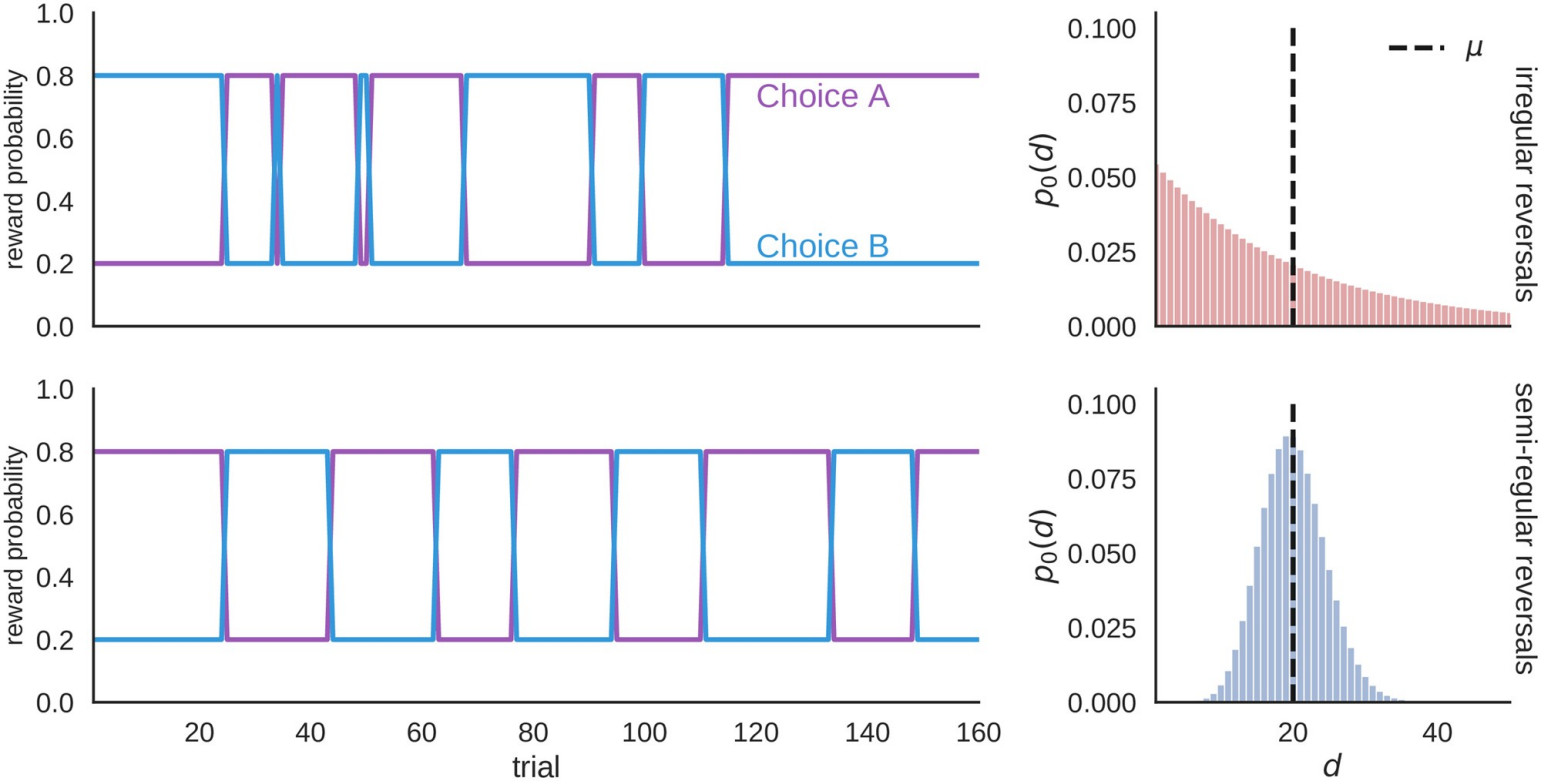
Example of a reversal schedule for simulated data. In the condition with irregular reversals, the between reversal intervals were sampled from a geometric distribution (top right plot), whereas in the condition with (semi)regular reversals, the intervals were sampled from the negative binomial distribution with variance *σ* = 20 (bottom right plot). In both cases the mean interval duration is set to *μ* = 20.

To illustrate the behavioral difference of different computational models we have modified the experimental setup and introduced two conditions in which reversals occur either at regular or irregular intervals, as shown in Fig 5. These two conditions allow us to make clear distinction between different behavioral models with respect to their behavior in the presence or absence of regularities.

In all simulated experiments we fixed the number of trials to *T* = 160 and the number of experimental blocks to *n* = 1000. Hence, each simulated agent repeated the experiment *n* times, where at the beginning of each experiment the free model parameters (besides mean and variance) of the ED-HMM based agents (IRI and RRI) were set to the following values

$$\begin{array}{c} \begin{array}{cc} {\tilde{a}}_{0}^{A} & =8;\;\;{\tilde{b}}_{0}^{A}=2,\\ {\tilde{a}}_{0}^{B} & =2;\;\;{\tilde{b}}_{0}^{B}=8,\\ V_{0}^{A} & =0;\;\;V_{0}^{B}=0,\\ \tilde{p}\left(d_{1}\right) & =p_{0}\left(d_{1}\right). \end{array} \end{array}$$ (32)

In other words, simulated agents have a loose initial knowledge of the underlying reward probabilities, but do not know the initial configuration of the task (whether the environment is initially in the reversal or the no-reversal state).

**Fig 6:**
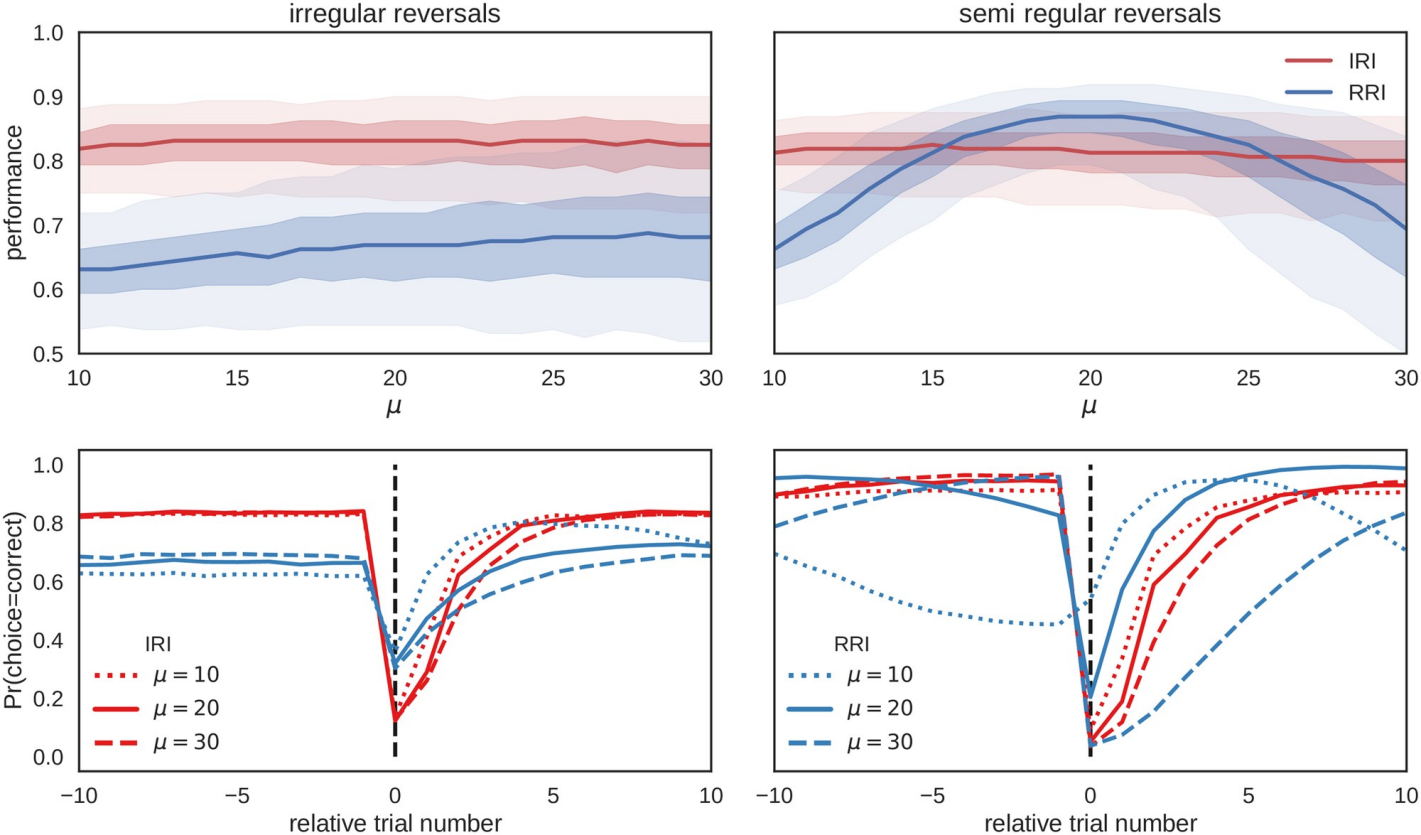
Performance as a function of the expected interval duration for ED-HMM based agents. The shaded regions show the performance quartiles estimated over *n* = 1000 simulated experiments, in two different task environments. Left: In the first environment, reversals occur at irregular intervals. The irregular reversal interval (IRI) agent (red) corresponds to the case where prior beliefs over interval duration follow a geometric distribution (see Eq (5)), with mean *μ* and variance *σ* = *μ*(*μ* − 1). This agent expects reversals at irregular intervals. The regular reversal agent (RRI) (blue) corresponds to the prior beliefs in the form of the negative binomial distribution, with mean *μ* and variance *σ* = *μ*. This agent expects reversals at (semi)regular intervals. The true distribution of between reversal intervals in both conditions is depicted in Fig 5. Right: Same as left plot but the environment has semi-regular intervals encoded by *μ* = 20 and *σ* = 20. As expected, we find that the RRI agent is highly sensitive to the misspecification of the expected interval duration *μ* and variability of interval durations in different conditions, while the IRI agent shows a much better performance in the irregular environment. The lower row shows the average probability of making a correct choice relative to the point of reversal, denoted by the vertical black dashed line.

Fig 6 shows the dependence of the agent’s performance on its prior beliefs about the between reversal interval in two different environments. We have defined the performance as the fraction of correct choices (the choice associated with the higher reward probability). The irregular environment corresponds to the interval duration drawn from the geometric distribution with mean *μ* = 20 and variance *σ* = *μ*(*μ* − 1), and the semi-regular environment corresponds to interval durations drawn from the negative binomial distribution with mean *μ* = 20 and variance *σ* = *μ*, as illustrated previously in Fig 5.

The IRI agent exhibits stable performance levels independent on the environment or a belief misspecification (incorrect mean interval duration, or incorrect variability around the mean). In contrast, the performance of the RRI model strongly depends on the correctness of the prior beliefs. These results make the rather intuitive point that if one is unfamiliar with the temporal structure of the environment, assigning high uncertainty to the expected state duration (as is the case for the IRI agent) ensures reasonably high levels of performance. However, if the environment changes at regular intervals, it is worthwhile to build an accurate representation of the temporal structure, as the performance levels can drastically increase (in this example from 81% to 87% of median performance levels).

**Fig 7:**
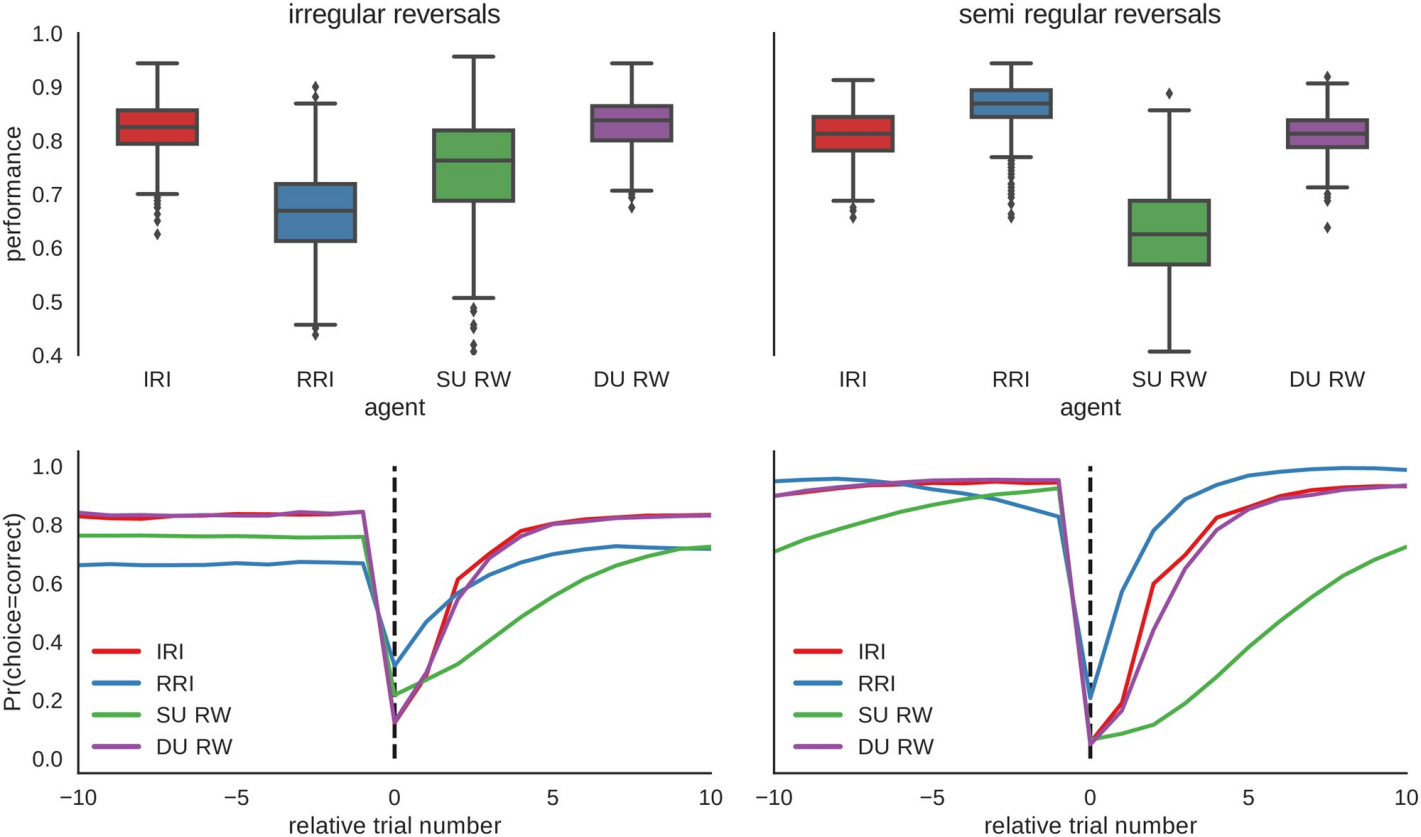
Performance distributions of all four behavioral models in two different environments. Here we compare the performance distributions of four different agents: irregular reversal interval (IRI) agent, regular reversal interval (RRI) agent, single-update Rescorla-Wagner(SU-RW) agent (Eq (1)) and dual-update Rescorla-Wagner (DU-RW) agent (Eq (2)). The free model parameters were fixed as follows (see main text for more details): (IRI) *μ* = 20, and *σ* = *μ*(*μ* − 1); (RRI) *μ* = 20, and *σ* = *μ*; (SU-RW) *α* = .25 and *κ* = 0; (DU-RW) *α* = .25 and *κ* = 1. Interestingly, the results show that the DU-RW agent achieves similar performance levels in both environments, with average responses closely following that of the IRI agent (see lower row). In contrast, the SU-RW agent shows only medium level performance in both environments with a significant increase of performance with irregular reversals. As in Fig 6, the RRI agent performs best, among all agents, when exposed to semi-regular reversals, but has the worst performance, among all agents, when exposed to irregular reversals.

Fig 7 shows the comparison of the performance distribution between four models; the probabilistic agents IRI and RRI, and the two reinforcement learning based agents with single update (SU-RW) and dual update (DU-RW) learning rules. The free model parameters (*α* and *κ*, see Table 1) of the SU-RW and DU-RW agents were fixed to those values that maximize the average performance levels in each environment. Importantly, when comparing the performance distribution of the DU-RW agent to the performance distribution of the IRI agent we find similar average performance per trial which is stable across environments. This finding suggests that in spite of subtle differences in learning rules (see Eqs (2) and (20)) the two agents generate very similar behavior.

**Fig 8:**
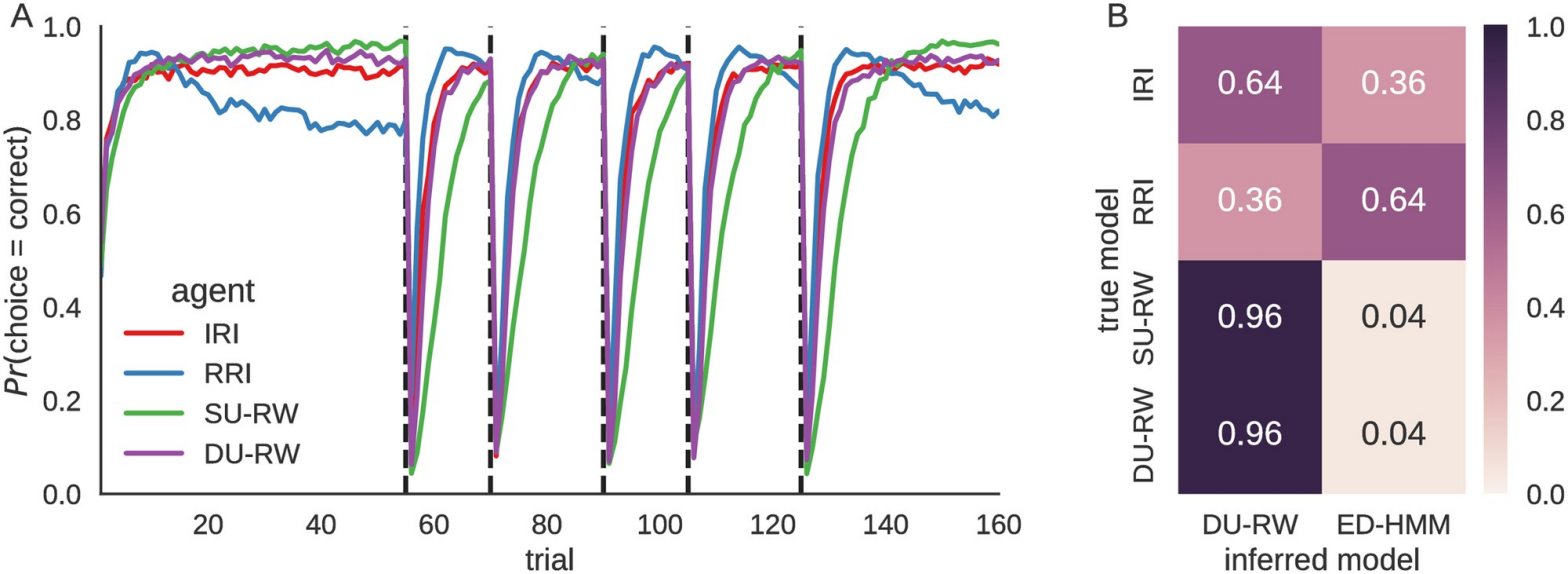
Agents performance on the experimental task and model comparison. **A:** Per trial performance (probability of making a correct choice) of four different agent types: irregular reversal interval (IRI) agent, regular reversal interval (RRI) agent, single-update Rescorla-Wagner(SU-RW) agent (Eq (1)) and dual-update Rescorla-Wagner (DU-RW) agent (Eq (2)). The free model parameters were fixed as follows: (IRI) *μ* = 20, and *σ* = *μ*(*μ* − 1); (RRI) *μ* = 20, and *σ* = 120; (SU-RW) *α* = .25 and *κ* = 0; (DU-RW) *α* = .25 and *κ* = 1. The parameters were fixed in a way that all models have comparable median performance levels (around 0.83). **B:** Confusion matrix showing the probability of assigning simulated behavior of different agents to the two different types of behavioral models. Note that as in Fig 7 the IRI and DU-RW agents generate very similar average responses across the experiment. This suggest a difficulty in distinguishing between these two type of models, which is also reflected in the confusion matrix that shows rather high mixing probability.

To investigate the accuracy of the procedure for model comparison which we described in Model fitting and model comparison, we have simulated behavior of the ED-HMM based and the reinforcement learning based agents on the experimental task, and applied the same model inversion procedure which we used for the analysis of behavioral data below. In Fig 8A we show the average performance per trial for each agent type estimated over *n* = 1000 repetitions of the experimental task. Fig 8B shows the probability of assigning behavior of each type of agents to the correct model type. The confusion matrix was estimated over *n* = 100 simulated experimental blocks, where in half of the experimental blocks ED-HMM based agents (IRI and RRI) were used to generate behavioral responses and in the other half of the experimental blocks the reinforcement learning agents (SU-RW and DU-RW) were used to generate behavioral responses. The rather high mixing probability of the confusion matrix suggests that the model comparison will have difficulties distinguishing properly between the models. Nevertheless, adjusting the experimental design to contain larger number of trials, that is, more data points for estimating posterior and model evidence, is likely to make model comparison more accurate.

### Data analysis

Here we will present model comparisons and model fitting based on the behavioral data of 22 participants. As the SU-RW model was shown in previous studies to provide a worse account for behavior than the DU-RW model [40], we do not expect the SU-RW model to explain the behavioral data better than the other models. Moreover, as our simulations indicate that the HMM and DU-RW models provide for comparable response patterns (see Fig 7), we will use only the DU-RW model as a reference model, as this model was used in a previous study based on the same data [39].

To circumvent a potential sensitivity of the model evidence to the specification of the prior distribution for the free model parameters, we have based our analysis on the posterior predictive model evidence (see Model fitting and model comparison for details). As the posterior predictive model evidence is estimated from the posterior distribution, rather than the prior (see Eq (28)), it is more robust to mis-specification of the prior, given a sufficient amount of data in the predictive sample. Hence, to estimate the posterior predictive evidence we have split the behavioral data for each participant into two sets. The first set, containing the initial 125 trials, that is the pre-reversal phase and the reversal phase of the experiment (see Fig 1), we used for estimating posterior distributions of free model parameters (see Table 1). Note that as this set contains all but the last reversal, it should provide sufficient information to constrain the posterior model parameters. The reversal phase of the experiment is the only period which participants can use to shape their beliefs about the time structure of reversals.

The second set, containing the last 35 trials, that is, the post-reversal phase (see Fig 1), we used for Bayesian model comparison and model validation. Critically, as no reversals are present in the post-reversal phase, this phase is especially suited for model selection: If participants believe that a reversal will occur at specific moments during the post-reversal phase this belief should be reflected in their behavior; e.g., they might change their choices in anticipation of a reversal.

**Fig 9:**
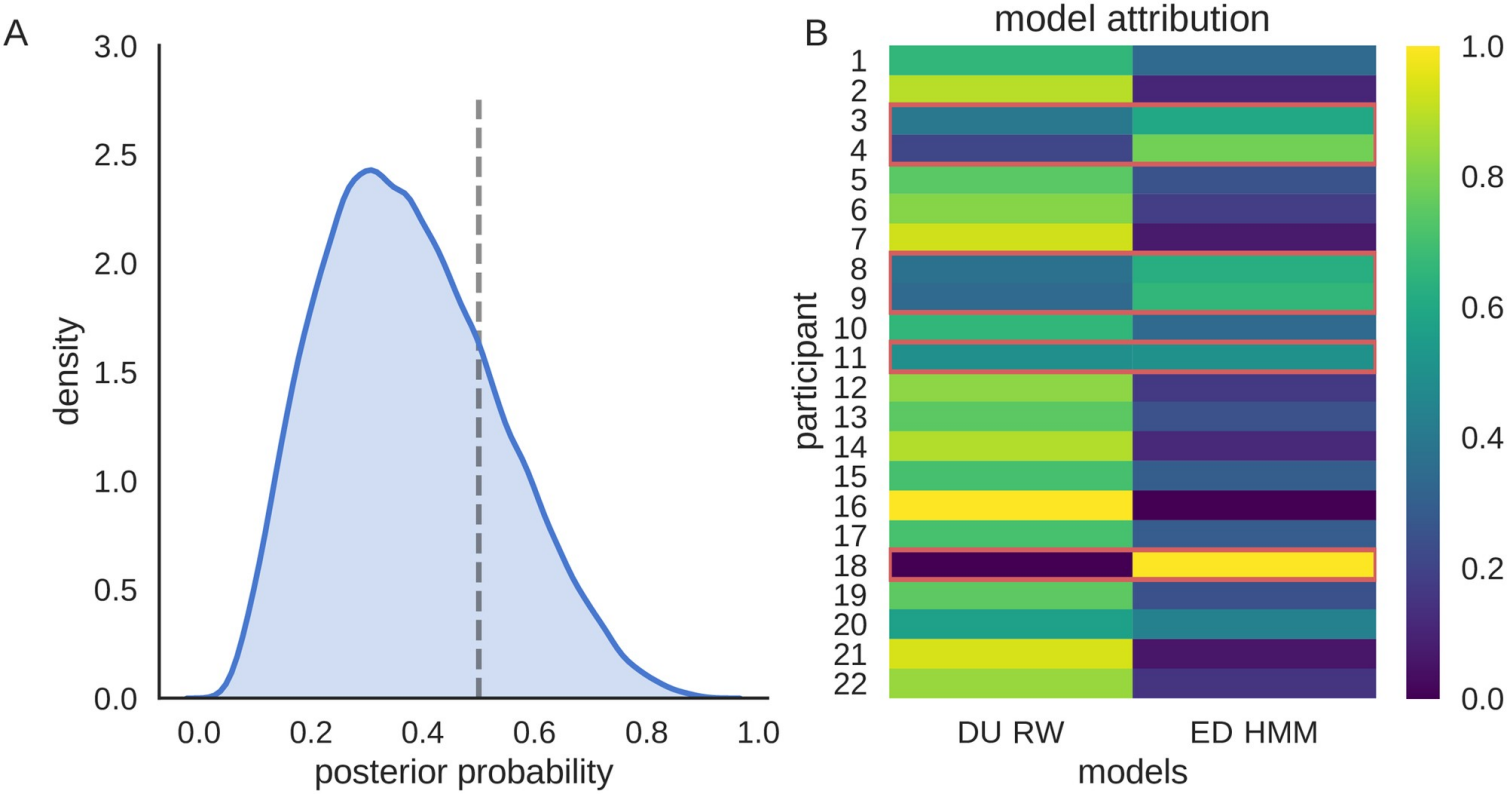
Bayesian model comparison. **A:** Posterior distribution of the frequency of the ED-HMM based model within the studied group of participants. The dashed line denotes the border at which both DU-RW and ED-HMM based models are equally likely. Hence, the probability mass on the right side of the dashed line (where ED-HMM has higher frequency within the population) corresponds to the exceedance probability (ep) of the ED-HMM based model (*ep* = 0.215). **B:** the color codes denote posterior model probability for each participant. The lighter the color the better the given model predicts behavior of the given participant during the post-reversal phase.

In Fig 9 we show the results at both the group level and the individual level, using Bayesian model comparison based on the posterior predictive model evidence. Although the group level results suggest substantial evidence in favor of the DU-RW model (see Fig 9A), we can still identify six participants for which we find higher model attribution (a participant specific posterior model probability) of the ED-HMM based model (see Fig 9B). The exact values of the predictive log model evidence (used for model comparison) and per subject model attribution are shown in S1 Table.

**Fig 10:**
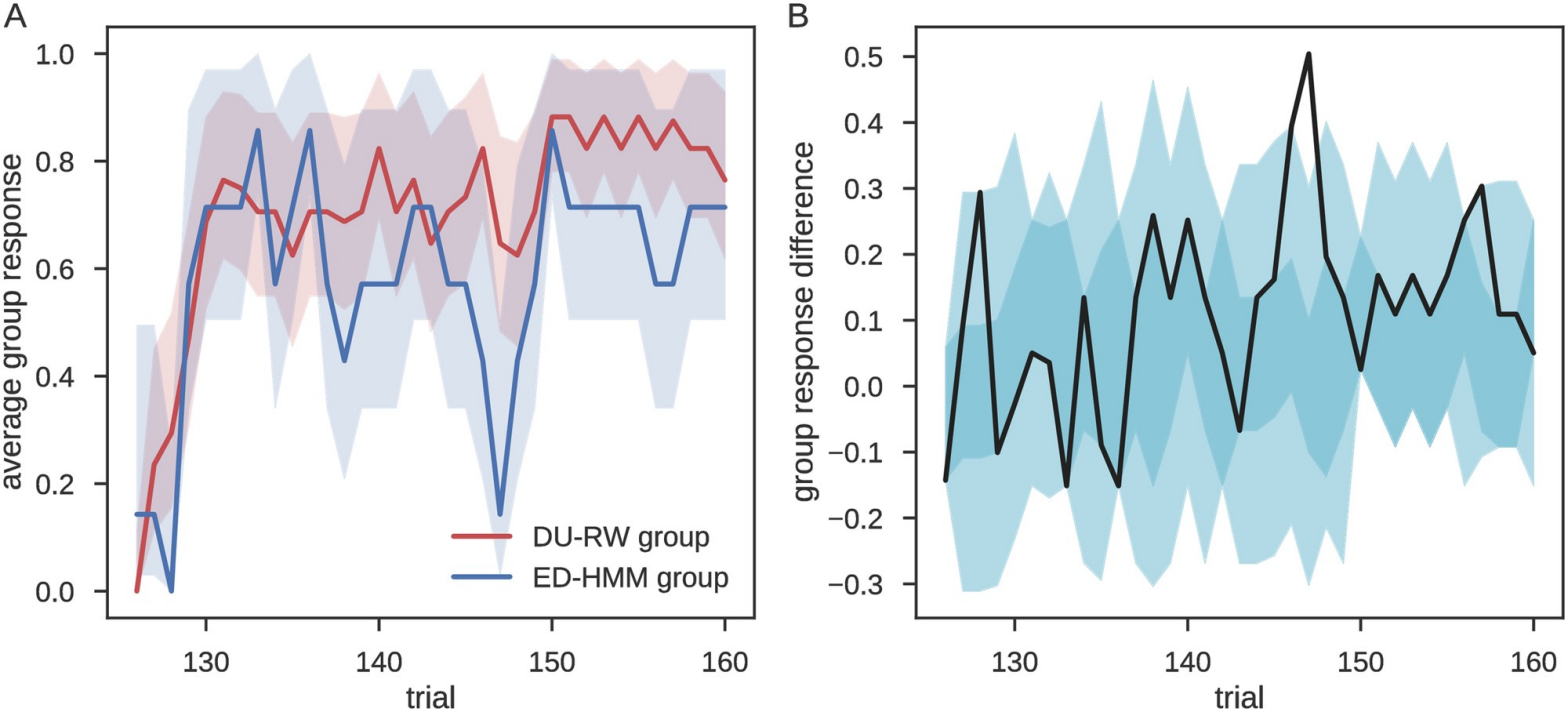
Mean participant responses during the post-reversal phase. **A**: Average response across the two groups of participants, where one group (red line) is best described by the DU-RW model, and the other group (blue line) by the ED-HMM. The shaded area marks the 90% Jeffreys interval. **B**: Difference between mean group responses (black line), where the shaded area marks the 5th-95th percentile (light cyan) and the 25th-75th percentile (dark cyan) of the group response differences obtained from 10^4^ random allocations of participants into groups of size 6 and 16. The peak of the response difference (at the 21st trial after the start of the post-reversal phase) lies well above the 95th percentile.

We next asked the question whether there is some systematic behavioral difference between these six and the remaining eighteen participants. Thus, we compared the averaged responses of participants belonging to the ED-HMM group and the participants belonging to the DU-RW group. In Fig 10 we show the choice probability averaged for each of the two groups. Tellingly, the ED-HMM group (blue line) exhibits a sudden switch toward the alternative choice approximately 20 trials after the start of post-reversal phase. Note that no reversals were induced in this phase. This result suggests that participants belonging to the group for which the ED-HMM model has higher posterior evidence behaved as if they were expecting another reversal 20 trials after the last one. This indicates that they have used the reversal phase to infer the reversal frequency and regularity.

**Fig 11:**
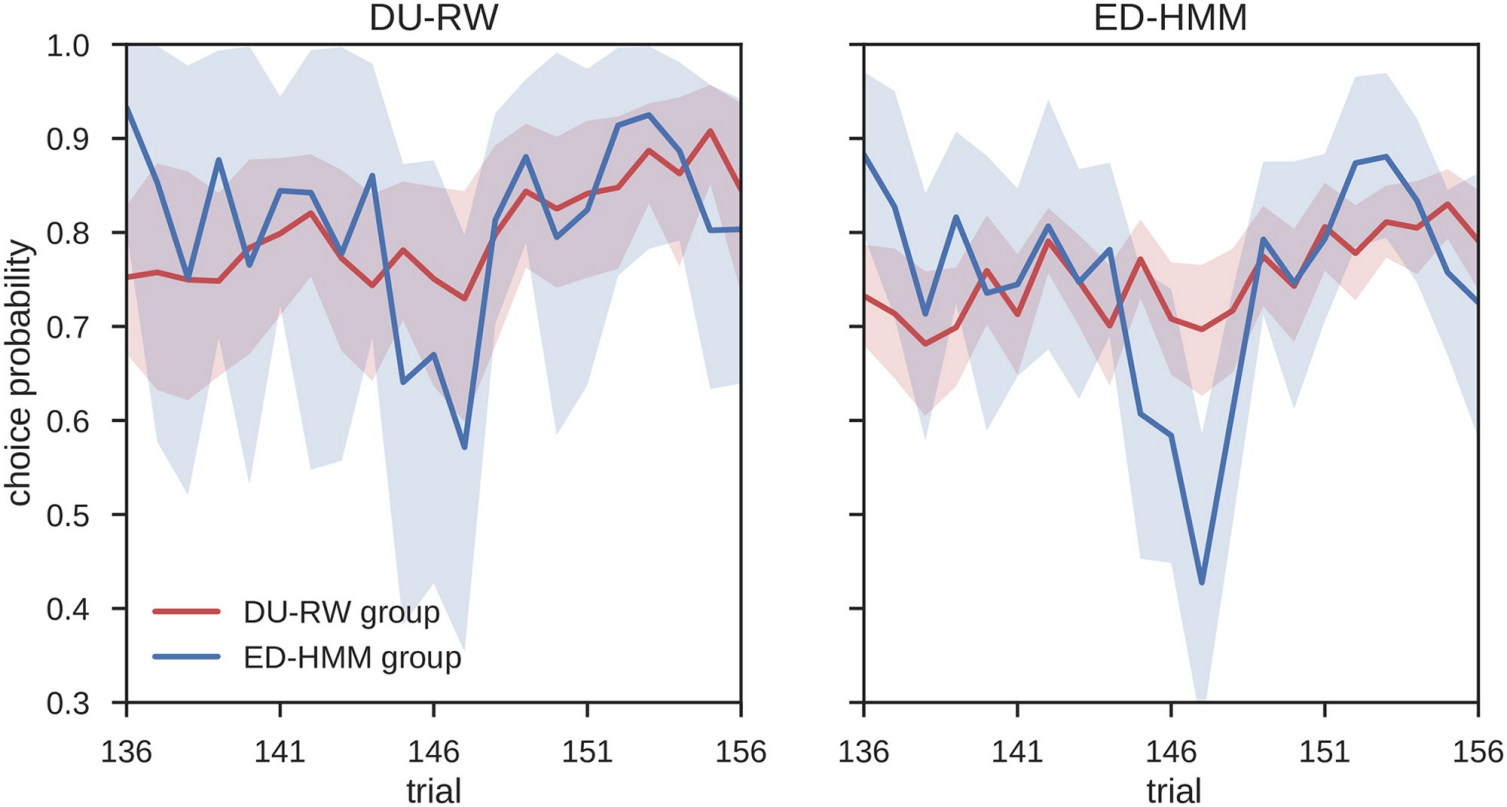
Model based mean response probabilities during the post-reversal phase. Response probabilities estimated from the DU-RW model (left) and ED-HMM model (right) and averaged over the two groups of participants: (blue line) The participants for which ED-HMM has higher model attribution, and (red line) participants for which DU-RW has higher model attribution. The shaded area corresponds to the 90% confidence interval of the group mean response probability.

If the ED-HMM model indeed captures this aspect of behavior better than the DU-RW model, we would expect to see a similar trend in the response probabilities estimated from the two behavioral models. For this we estimated the response probability for each participant under each of the two models and averaged responses according to group. In Fig 11 we show the between model comparison of the estimated response probabilities for the two groups of participants. Although the mean response of the DU-RW model also shows a trough towards the alternative choice (when conditioned on the participants in the ED-HMM group), we see a much wider excursion in the mean response probability obtained using the ED-HMM, explaining its higher predictive model evidence for this group of participants. Still, it is important to note that the presence of the trough in the mean response probability of both models suggest that the change of response probability towards the alternative choice was driven by the specific sequence of outcomes that participants observed during this time window of the post-reversal phase. In other words, it seems that the participants that were assigned to the ED-HMM group were sensitive to a short sequence of negative outcomes, as if they were expecting another reversal during the post-reversal phase.

**Fig 12:**
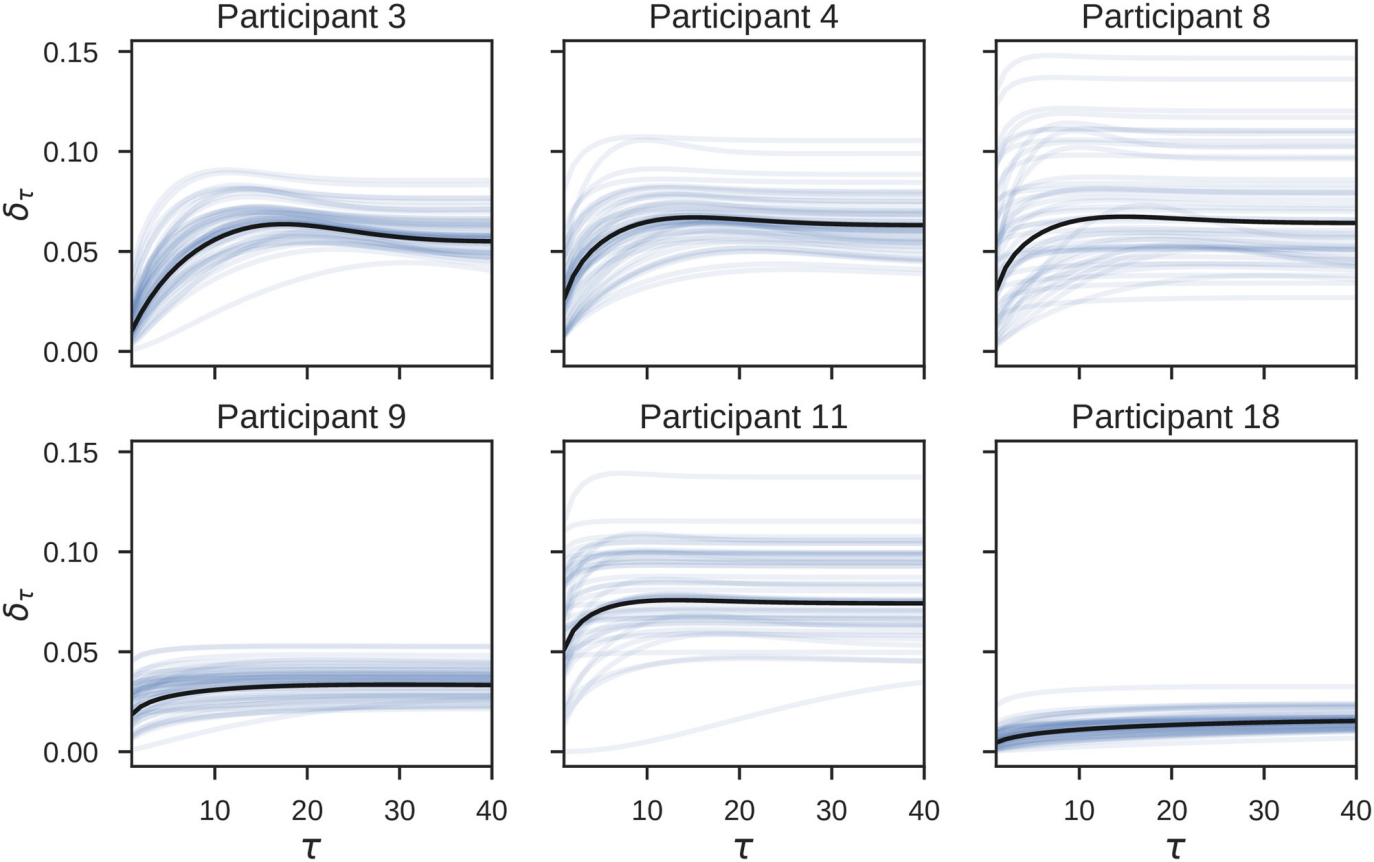
Posterior estimate of the expected reversal probability. The participants’ specific beliefs about the probability of reversal within the post-reversal phase, for all participants for which the ED-HMM model had higher posterior model evidence. The reversal probability is fully characterized by two parameters *δ*, and *r* (see Eqs (8) and (9)). Each light blue line corresponds to the trajectory obtained as a single sample from the posterior estimates over *δ* and *r*, whereas black lines correspond to the trajectory obtained from the mean posterior parameter estimates *μ*_*r*_ and *μ*_*δ*_.

In Fig 12 we show the posterior estimates of the expected reversal probability *δ*_*τ*_ for all participants of the ED-HMM group. The expected reversal probability was estimated from State durations and corresponds to the measure illustrated in Fig 4. Note that for four out of the six participants we find the peak of the reversal probability to fall before *τ* = 20, that is, before the 20th trial within the post-reversal phase. For the other two we see a rather flat trajectory which suggests that these subjects behave close to the IRI agents. Although these results seem to be in contrast to our previous findings (that these six participants anticipated the change on the 20th trial of the post reversal phase), the expected reversal probability does not correspond to the expected response probability for a specific participant. The relation between these two quantities is non-linear and is also shaped by the sequence of outcomes.

## Discussion

The ability to represent complex temporal structures and to use these representations in everyday decision-making is one of the key features of human adaptive behavior [62]. However, the computational mechanisms that underlie these aspect of behavior are still poorly understood. Here we proposed a novel behavioral model which utilizes an explicit representation of state durations for making decisions. This model allowed us to investigate how beliefs about the temporal structure of latent states in the environment influence the decision-making process.

As a demonstration of the applicability of the proposed model to behavioral data we have used a reversal learning task, in which reversals followed a pre-specified temporal pattern. We have illustrated several properties of the novel model, using both synthetic data for model validation and experimental data for a proof-of-concept demonstration.

Fitting the model to behavioral data allowed us to infer individual beliefs in temporal regularities and identify participants who rely on the latent reversal schedule used in the experimental task. Interestingly, we found that slightly more than one fourth of the participants showed evidence of using temporal expectations when making choices during the post-reversal phase of the experiment. Although the majority of participants behaved as if they did not use any temporal regularity in the sequence of the reversals, this result is not too surprising because participants were only exposed to five reversals during the whole experiment. We expect that prolonged exposure to reversals would allow a larger number of participants to correctly learn the latent task structure, as it was recently demonstrated in [28].

We anticipate that the proposed model can bring novel insights into our understanding of the interplay between the subjective representation of temporal task structure and decision making. The relevance of accurately predicting the moments of change in our everyday lives is reflected by the fact that humans exhibit a strong bias towards expecting timed changes in various tasks. This behavior can even persist when exposed to a series of unpredictable events, e.g., during gambling [63] or trading on financial markets [64]. Hence, it may not be too surprising that a firm prior belief in structured and predictable changes influences performance and may lead to a reduced performance in cases when these beliefs are incorrect (see Figs 6 and 7). Such over-confidence in temporally structured dynamics might also explain why human behavior is found to deviate from the fully-rational Bayesian model on similar reversal learning tasks [48].

### Related work

The key component of the proposed behavioral model is its conceptualization as an explicit duration hidden Markov models ED-HMM [30], which involves an explicit representation of the between reversal intervals as the hidden structural variable. This representation results in an anticipation of specific moments of reversals. Such anticipation would be clearly advantageous for an agent, as it enables faster behavioral adaptation in cases when reversals actually do occur in a (semi)regular manner.

The ED-HMM belong to more general group of hidden semi-Markov models which are often applied to the analysis of non-stationary time series [65–68]. In the context of decision making the semi-Markov formalism allows for temporal structuring of behavioral policies [69]. Importantly, semi-Markov dynamics was also applied to temporal difference learning to account for dopamine activity in cases when the timing between action and reward is varied between trials [70].

The proposed model builds upon recent approaches to model behavior in changing environments [11, 71, 72] and can be seen as a direct extension of hidden Markov models (HMM) which were applied to reversal learning tasks before [29, 38, 46–48]. In previous works, the HMM were used to identify the moment of reversal, changes in the beliefs about reversal probability, and the most likely moment in which agents reversed their behavior. This was crucial for understanding the effects of dopamine modulation on the underlying inference and consequently behavior. Although it is out of the scope of the present paper, it is possible to perform backward inference with hidden semi-Markov models (HSMM), hence identifying the most likely moments of reversal in the past. Furthermore, we will explore in the future possible learning rules for parameters of the prior beliefs *p*_0_(*d*), similar to the work of [73]. Such extension would make the models also suitable for addressing questions related to changes in prior beliefs of state durations.

In recent years, reinforcement learning models have found multiple applications in studies relying on a reversal learning task. For example, the classical Rescorla-Wagner model [74], the dual update extension of the Rescorla-Wagner model (as described in the present paper) [39, 40], or models separating the prediction error signals on positive and negative prediction errors [75]. As we have shown here a reinforcement learning model generates behavior very similar to a probabilistic counterpart in relatively simple settings of the reversal learning task. Hence, we would expect that additional extensions of the considered dual update RW model could make the behavior of the reinforcement learning even more similar to the probabilistic model introduced here.

Still, we can point out several advantages of probabilistic models of behavior over reinforcement learning models, in the context of decision making under uncertainty and in dynamic environments. The probabilistic modeling approach allows for a principle way of mapping complex knowledge about spatio-temporal task structure into a relatively simple set of learning rules (as demonstrated here). In turn this provides clear functional interpretation of various prediction error signals, and corresponding adaptive learning rates, which are typically difficult to derive or motivate within the context of classical reinforcement learning. Specifically, we would say that prescribing to a probabilistic modeling approach is crucial for understanding interaction between representation of temporal structure and decision making.

### Interval timing

The sense of time and time representation at a neuronal level has been the focus of numerous studies in the past [76–82]. Studies investigated how neuronal circuitry might implement a robust internal clock [83], and how such an internal clock can be utilized to estimate time elapsed between consequent events (e.g., between two tones). Although not described here, behavior in such and similar tasks can be modelled using the formalism of the hidden semi-Markov models. Estimating elapsed time between events (here, reversals) can be obtained using a backward inference process which provides an estimate on the most likely moment of the previous reversal. Nevertheless, it is an open question if the neural circuitry that explains behavior related to interval timing, which involves sub-second time scales, can also be useful to understand behavior which requires a temporal reasoning over much longer time scale. This is of specific interest for understanding how the brain makes accurate predictions about the expected moment of reward, and how this information is used to construct timed prediction error signals [84, 85].

### Variants of the reversal learning task

The reversal learning task has been shown to be especially useful for behavioral phenotyping, e.g. in areas of executive control [86–90]; for quantifying individual proneness towards impulsive and compulsiveness [91]; and defining individual vulnerability towards addiction [92] and other psychiatric disorders [35, 93, 94]. The experimental paradigms associated with a reversal learning task can differ substantially from the design presented here (see Fig 1). For example, the probability of reward and punishment for different choices could be correlated to some degree [47, 95], or the reversal schedule can be made dependent on the number of correct choices [96–98]. It is relatively straightforward to adapt the proposed model to any of these variants. For example, when the reversal schedule depends on the number of previously correct choices, one can make the duration transitions dependent on the belief that the previous choice was correct. This would relate state durations to the beliefs about the number of correct choices since the last reversal. Thus, we believe that in a combination with the proposed model, the reversal learning task opens a wide range of opportunities for linking anomalous beliefs in the time domain to different cognitive disorders.

### Relevance for understanding psychiatric disorders

Distortions in the temporal organization of cognition and behavior have been implicated, for a long time, in a wide range of psychiatric conditions, perhaps most prominently in attention-deficit hyperactivity disorder, autism and schizophrenia [99]. Hence, the proposed model might help to integrate findings of patients’ aberrant behavior in simple time estimation tasks (e.g., a deficit to reproduce durations [100–102] with their known deficits in the decision-making domain [103], such as, in variants of the reversal learning task.

Over the recent years, the question of how people apply their knowledge about the underlying structure of the environment in their decision-making has been widely investigated and discussed. Studies have come to the rather unspecific notion that a reduction in model-based control based on using environmental structure is ubiquitous across different psychiatric symptoms and diagnoses [104, 105]. We believe that the model at hand could be a starting point for developing a set of tasks particularly appropriate for refining this notion.

For example, Bayesian theoretical accounts have conceptualized addictive behavior as decision-making based on an aberrant model of the world [106]. Both animal and human studies suggest that addicted patients might have a specific deficit to infer or simulate statistical regularities in the environment [40, 107, 108]. Such deficits suggest limitations in their ability to infer regularities, which consequently is observed as suboptimal decision-making.

Reversal learning deficits and impairments in model-based control have also been shown in patients suffering from obsessive compulsive disorder (OCD) [109, 110]. A subgroup of OCD patients suffers from an obsession for symmetry and organization. Similarly, obsessive compulsive personality disorder (OCPD) patients show an exaggerated focus on order and symmetry as well as rule-bound traits. Given this clinical picture, it is interesting to speculate that beliefs about regularities in the environment might differ between OCD/OCPD patients and controls. A reduced ability to infer and represent irregular changes in the environment might lead to suboptimal decision-making and distress in OCD/OCPD patients in the presence of irregular changes.

Finally, recent studies have demonstrated that schizophrenia patients may be characterized by an “over-dominance” of an internal model [111] (e.g., in the framework presented here, overrating regularities in an irregular environment). When such an internal model is detached from the external input from the world (i.e., the true regularity structure), this mismatch might be involved in the development of psychotic states (in paranoia, delusions, hallucinations).

### Conclusion

In summary, we have presented a novel probabilistic model for understanding human decision making behavior in changing environments. The proposed model is based on the explicit duration hidden Markov model, which forms a special case of more general hidden semi-Markov models. The presented results, obtained from both simulated behavior and the model-based analysis of behavioral data, suggest concrete applications of the proposed model in understanding human behavior in changing environments. Most notably, it might be possible to link the quality of the underlying representation of temporal task structure to behavior, thereby opening new directions for cognitive phenotyping.

## Supporting information

S1 Table Model evidence and model attribution. Subject specific values of the estimated log posterior predictive model evidence (PPLME) and the corresponding model attribution (see Model fitting and model comparison for details).
(PDF)

## References

[1] Grondin S. Timing and time perception: A review of recent behavioral and neuroscience findings and theoretical directions. Attention Perception, & Psychophysics. 2010;72(3):561–582. 10.3758/APP.72.3.561 20348562

[2] Buhusi CV, Meck WH. What makes us tick? Functional and neural mechanisms of interval timing. Nature Reviews Neuroscience. 2005;6(10):755–765. 10.1038/nrn1764 16163383

[3] Howard MW, Eichenbaum H. Time and space in the hippocampus. Brain research. 2015;1621:345–354. 10.1016/j.brainres.2014.10.069 25449892 PMC4426245

[4] Meck WH, Penney TB, Pouthas V. Cortico-striatal representation of time in animals and humans. Current opinion in neurobiology. 2008;18(2):145–152. 10.1016/j.conb.2008.08.002 18708142

[5] Harrington DL, Boyd LA, Mayer AR, Sheltraw DM, Lee RR, Huang M, et al Neural representation of interval encoding and decision making. Cognitive Brain Research. 2004;21(2):193–205. 10.1016/j.cogbrainres.2004.01.010 15464351

[6] Finnerty GT, Shadlen MN, Jazayeri M, Nobre AC, Buonomano DV. Time in cortical circuits. Journal of Neuroscience. 2015;35(41):13912–13916. 10.1523/JNEUROSCI.2654-15.2015 26468192 PMC4604229

[7] Wilson RC, Takahashi YK, Schoenbaum G, Niv Y. Orbitofrontal cortex as a cognitive map of task space. Neuron. 2014;81(2):267–279. 10.1016/j.neuron.2013.11.005 24462094 PMC4001869

[8] Gershman SJ, Niv Y. Learning latent structure: carving nature at its joints. Current opinion in neurobiology. 2010;20(2):251–256. 10.1016/j.conb.2010.02.008 20227271 PMC2862793

[9] O’Doherty JP, Dayan P, Friston K, Critchley H, Dolan RJ. Temporal difference models and reward-related learning in the human brain. Neuron. 2003;38(2):329–337. 10.1016/S0896-6273(03)00169-7 12718865

[10] O’Reilly JX. Making predictions in a changing world—inference uncertainty, and learning. Frontiers in Neuroscience. 2013;7 10.3389/fnins.2013.00105 23785310 PMC3682109

[11] Payzan-LeNestour E, Bossaerts P. Risk, unexpected uncertainty, and estimation uncertainty: Bayesian learning in unstable settings. PLoS computational biology. 2011;7(1):e1001048 10.1371/journal.pcbi.1001048 21283774 PMC3024253

[12] Pearson JM, Heilbronner SR, Barack DL, Hayden BY, Platt ML. Posterior cingulate cortex: adapting behavior to a changing world. Trends in Cognitive Sciences. 2011;15(4):143–151. 10.1016/j.tics.2011.02.002 21420893 PMC3070780

[13] Ghahremani DG, Monterosso J, Jentsch JD, Bilder RM, Poldrack RA. Neural Components Underlying Behavioral Flexibility in Human Reversal Learning. Cerebral Cortex. 2009;20(8):1843–1852. 10.1093/cercor/bhp247 19915091 PMC2901019

[14] Ballard I, Miller EM, Piantadosi ST, Goodman ND, McClure SM. Beyond Reward Prediction Errors: Human Striatum Updates Rule Values During Learning. Cerebral Cortex. 2017; p. 1–11. 29040494 10.1093/cercor/bhx259 PMC6685076

[15] Kolossa A, Kopp B, Fingscheidt T. A computational analysis of the neural bases of Bayesian inference. NeuroImage. 2015;106:222–237. 10.1016/j.neuroimage.2014.11.007. 25462794

[16] Meyniel F, Schlunegger D, Dehaene S. The sense of confidence during probabilistic learning: A normative account. PLoS computational biology. 2015;11(6):e1004305 10.1371/journal.pcbi.1004305 26076466 PMC4468157

[17] Diaconescu AO, Mathys C, Weber LA, Daunizeau J, Kasper L, Lomakina EI, et al Inferring on the intentions of others by hierarchical Bayesian learning. PLoS computational biology. 2014;10(9):e1003810 10.1371/journal.pcbi.1003810 25187943 PMC4154656

[18] Iglesias S, Mathys C, Brodersen KH, Kasper L, Piccirelli M, den Ouden HE, et al Hierarchical prediction errors in midbrain and basal forebrain during sensory learning. Neuron. 2013;80(2):519–530. 10.1016/j.neuron.2013.09.009 24139048

[19] Payzan-LeNestour E, Dunne S, Bossaerts P, O’Doherty JP. The Neural Representation of Unexpected Uncertainty during Value-Based Decision Making. Neuron. 2013;79(1):191–201. 10.1016/j.neuron.2013.04.037. 23849203 PMC4885745

[20] Ide JS, Shenoy P, Angela JY, Chiang-Shan RL. Bayesian prediction and evaluation in the anterior cingulate cortex. Journal of Neuroscience. 2013;33(5):2039–2047. 10.1523/JNEUROSCI.2201-12.2013 23365241 PMC3711643

[21] Nassar MR, Rumsey KM, Wilson RC, Parikh K, Heasly B, Gold JI. Rational regulation of learning dynamics by pupil-linked arousal systems. Nature neuroscience. 2012;15(7):1040–1046. 10.1038/nn.3130 22660479 PMC3386464

[22] Friston K. The free-energy principle: a unified brain theory? Nature Reviews Neuroscience. 2010;11(2):127 10.1038/nrn2787 20068583

[23] Knill DC, Pouget A. The Bayesian brain: the role of uncertainty in neural coding and computation. TRENDS in Neurosciences. 2004;27(12):712–719. 10.1016/j.tins.2004.10.007 15541511

[24] Cravo AM, Rohenkohl G, Santos KM, Nobre AC. Temporal anticipation based on memory. Journal of cognitive neuroscience. 2017;29(12):2081–2089. 10.1162/jocn_a_01172 28777060 PMC5884434

[25] van Ede F, Niklaus M, Nobre AC. Temporal expectations guide dynamic prioritization in visual working memory through attenuated *α* oscillations. Journal of Neuroscience. 2017;37(2):437–445. 10.1523/JNEUROSCI.2272-16.2016 28077721 PMC5242399

[26] Auksztulewicz R, Friston KJ, Nobre AC. Task relevance modulates the behavioural and neural effects of sensory predictions. PLoS biology. 2017;15(12):e2003143 10.1371/journal.pbio.2003143 29206225 PMC5730187

[27] Rohenkohl G, Gould IC, Pessoa J, Nobre AC. Combining spatial and temporal expectations to improve visual perception. Journal of vision. 2014;14(4):8–8. 10.1167/14.4.8 24722562 PMC3983934

[28] Vilà-Balló A, Mas-Herrero E, Ripollés P, Simó M, Miró J, Cucurell D, et al Unraveling the role of the hippocampus in reversal learning. Journal of Neuroscience. 2017;37(28):6686–6697. 10.1523/JNEUROSCI.3212-16.2017 28592695 PMC6596552

[29] Costa VD, Tran VL, Turchi J, Averbeck BB. Reversal learning and dopamine: a bayesian perspective. Journal of Neuroscience. 2015;35(6):2407–2416. 10.1523/JNEUROSCI.1989-14.2015 25673835 PMC4323525

[30] Dewar M, Wiggins C, Wood F. Inference in Hidden Markov Models with Explicit State Duration Distributions. IEEE Signal Processing Letters. 2012;19(4):235–238. 10.1109/LSP.2012.2184795

[31] Yu SZ. Hidden semi-Markov models. Artificial intelligence. 2010;174(2):215–243. 10.1016/j.artint.2009.11.011

[32] Attias H. Planning by Probabilistic Inference. In: Proceedings of the Ninth International Workshop on Artificial Intelligence and Statistics, AISTATS 2003, Key West, Florida, USA, January 3-6, 2003; 2003. Available from: http://research.microsoft.com/en-us/um/cambridge/events/aistats2003/proceedings/206.pdf.

[33] Botvinick M, Toussaint M. Planning as inference. Trends in Cognitive Sciences. 2012;16(10):485–488. 10.1016/j.tics.2012.08.006 22940577

[34] Friston K, Rigoli F, Ognibene D, Mathys C, Fitzgerald T, Pezzulo G. Active inference and epistemic value. Cognitive Neuroscience. 2015;6(4):187–214. 10.1080/17588928.2015.1020053 25689102

[35] Clarke HF. Prefrontal Serotonin Depletion Affects Reversal Learning But Not Attentional Set Shifting. Journal of Neuroscience. 2005;25(2):532–538. 10.1523/JNEUROSCI.3690-04.2005 15647499 PMC6725478

[36] den Ouden HE, Daw ND, Fernandez G, Elshout JA, Rijpkema M, Hoogman M, et al Dissociable effects of dopamine and serotonin on reversal learning. Neuron. 2013;80(4):1090–1100. 10.1016/j.neuron.2013.08.030 24267657

[37] Behrens TEJ, Woolrich MW, Walton ME, Rushworth MFS. Learning the value of information in an uncertain world. Nature Neuroscience. 2007;10(9):1214–1221. 10.1038/nn1954 17676057

[38] Hampton AN, Bossaerts P, O’doherty JP. The role of the ventromedial prefrontal cortex in abstract state-based inference during decision making in humans. Journal of Neuroscience. 2006;26(32):8360–8367. 10.1523/JNEUROSCI.1010-06.2006 16899731 PMC6673813

[39] Reiter AM, Heinze HJ, Schlagenhauf F, Deserno L. Impaired flexible reward-based decision-making in binge eating disorder: evidence from computational modeling and functional neuroimaging. Neuropsychopharmacology. 2017;42(3):628–637. 10.1038/npp.2016.95 27301429 PMC5240187

[40] Reiter AM, Deserno L, Kallert T, Heinze HJ, Heinz A, Schlagenhauf F. Behavioral and neural signatures of reduced updating of alternative options in alcohol-dependent patients during flexible decision-making. Journal of Neuroscience. 2016;36(43):10935–10948. 10.1523/JNEUROSCI.4322-15.2016 27798176 PMC6705653

[41] Li J, Schiller D, Schoenbaum G, Phelps EA, Daw ND. Differential roles of human striatum and amygdala in associative learning. Nature neuroscience. 2011;14(10):1250–1252. 10.1038/nn.2904 21909088 PMC3268261

[42] O’Doherty JP, Hampton A, Kim H. Model-Based fMRI and Its Application to Reward Learning and Decision Making. Annals of the New York Academy of Sciences. 2007;1104(1):35–53. 10.1196/annals.1390.022 17416921

[43] Lohrenz T, McCabe K, Camerer CF, Montague PR. Neural signature of fictive learning signals in a sequential investment task. Proceedings of the National Academy of Sciences. 2007;104(22):9493–9498. 10.1073/pnas.0608842104 PMC1876162 17519340

[44] Dymarski P. Hidden Markov models, theory and applications. InTechOpen; 2011.

[45] Murphy KP. Hidden semi-markov models (hsmms). unpublished notes. 2002;2.

[46] Jang AI, Costa VD, Rudebeck PH, Chudasama Y, Murray EA, Averbeck BB. The role of frontal cortical and medial-temporal lobe brain areas in learning a bayesian prior belief on reversals. Journal of Neuroscience. 2015;35(33):11751–11760. 10.1523/JNEUROSCI.1594-15.2015 26290251 PMC4540808

[47] Schlagenhauf F, Huys QJ, Deserno L, Rapp MA, Beck A, Heinze HJ, et al Striatal dysfunction during reversal learning in unmedicated schizophrenia patients. Neuroimage. 2014;89:171–180. 10.1016/j.neuroimage.2013.11.034 24291614 PMC3991847

[48] Hu Y, Kayaba Y, Shum M. Nonparametric learning rules from bandit experiments: The eyes have it! Games and Economic Behavior. 2013;81:215–231. 10.1016/j.geb.2013.05.003

[49] Yu SZ, Kobayashi H. An efficient forward-backward algorithm for an explicit-duration hidden Markov model. IEEE signal processing letters. 2003;10(1):11–14. 10.1109/LSP.2002.806705

[50] Vaseghi S. State duration modelling in hidden Markov models. Signal processing. 1995;41(1):31–41. 10.1016/0165-1684(94)00088-H

[51] Blei DM, Kucukelbir A, McAuliffe JD. Variational Inference: A Review for Statisticians. Journal of the American Statistical Association. 2017;112(518):859–877. 10.1080/01621459.2017.1285773

[52] Wainwright MJ, Jordan MI, et al Graphical models, exponential families, and variational inference. Foundations and Trends^®^ in Machine Learning. 2008;1(1–2):1–305.

[53] Beal MJ, et al. Variational algorithms for approximate Bayesian inference. university of London London; 2003.

[54] Friston K, Herreros I. Active inference and learning in the cerebellum. Neural computation. 2016 10.1162/NECO_a_00863 27391681

[55] Friston K, Rigoli F, Ognibene D, Mathys C, Fitzgerald T, Pezzulo G. Active inference and epistemic value. Cognitive neuroscience. 2015;6(4):187–214. 10.1080/17588928.2015.1020053 25689102

[56] Schönberg T, Daw ND, Joel D, O’Doherty JP. Reinforcement learning signals in the human striatum distinguish learners from nonlearners during reward-based decision making. Journal of Neuroscience. 2007;27(47):12860–12867. 10.1523/JNEUROSCI.2496-07.2007 18032658 PMC6673291

[57] Daw ND, Doya K. The computational neurobiology of learning and reward. Current opinion in neurobiology. 2006;16(2):199–204. 10.1016/j.conb.2006.03.006 16563737

[58] Salvatier J, Wiecki TV, Fonnesbeck C. Probabilistic programming in Python using PyMC3. PeerJ Computer Science. 2016;2:e55 10.7717/peerj-cs.55 PMC10495961 37705656

[59] Kucukelbir A, Tran D, Ranganath R, Gelman A, Blei DM. Automatic Differentiation Variational Inference. Journal of Machine Learning Research. 2017;18(14):1–45.

[60] Rigoux L, Stephan KE, Friston KJ, Daunizeau J. Bayesian model selection for group studies—revisited. Neuroimage. 2014;84:971–985. 10.1016/j.neuroimage.2013.08.065 24018303

[61] Gelman A, Hwang J, Vehtari A. Understanding predictive information criteria for Bayesian models. Statistics and Computing. 2014;24(6):997–1016. 10.1007/s11222-013-9416-2

[62] Muller T, Nobre AC. Perceiving the passage of time: neural possibilities. Annals of the New York Academy of Sciences. 2014;1326(1):60–71. 10.1111/nyas.12545 25257798 PMC4336553

[63] Croson R, Sundali J. The Gambler’s Fallacy and the Hot Hand: Empirical Data from Casinos. Journal of Risk and Uncertainty. 2005;30(3):195–209. 10.1007/s11166-005-1153-2

[64] Rabin M, Vayanos D. The Gambler’s and Hot-Hand Fallacies: Theory and Applications. Review of Economic Studies. 2010;77(2):730–778. 10.1111/j.1467-937X.2009.00582.x

[65] Tokdar S, Xi P, Kelly RC, Kass RE. Detection of bursts in extracellular spike trains using hidden semi-Markov point process models. Journal of computational neuroscience. 2010;29(1-2):203–212. 10.1007/s10827-009-0182-2 19697116

[66] Gales M, Young S, et al The application of hidden Markov models in speech recognition. Foundations and Trends^®^ in Signal Processing. 2008;1(3):195–304. 10.1561/2000000004

[67] Faisan S, Thoraval L, Armspach JP, Metz-Lutz MN, Heitz F. Unsupervised learning and mapping of active brain functional MRI signals based on hidden semi-Markov event sequence models. IEEE transactions on medical imaging. 2005;24(2):263–276. 10.1109/TMI.2004.841225 15707252

[68] Duong TV, Bui HH, Phung DQ, Venkatesh S. Activity recognition and abnormality detection with the switching hidden semi-markov model. In: Computer Vision and Pattern Recognition, 2005. CVPR 2005. IEEE Computer Society Conference on. vol. 1. IEEE; 2005. p. 838–845.

[69] Bradtke SJ, Duff MO. Reinforcement learning methods for continuous-time Markov decision problems. In: Advances in neural information processing systems; 1995. p. 393–400.

[70] Daw ND, Courville AC, Touretzky DS. Timing and partial observability in the dopamine system. In: Advances in neural information processing systems; 2003. p. 99–106.

[71] Mathys C, Daunizeau J, Friston KJ, Stephan KE. A Bayesian foundation for individual learning under uncertainty. Frontiers in human neuroscience. 2011;5:39 10.3389/fnhum.2011.00039 21629826 PMC3096853

[72] Nassar MR, Wilson RC, Heasly B, Gold JI. An approximately Bayesian delta-rule model explains the dynamics of belief updating in a changing environment. Journal of Neuroscience. 2010;30(37):12366–12378. 10.1523/JNEUROSCI.0822-10.2010 20844132 PMC2945906

[73] Wilson RC, Nassar MR, Gold JI. Bayesian online learning of the hazard rate in change-point problems. Neural computation. 2010;22(9):2452–2476. 10.1162/NECO_a_00007 20569174 PMC2966286

[74] Roesch MR, Esber GR, Li J, Daw ND, Schoenbaum G. Surprise! Neural correlates of Pearce–Hall and Rescorla–Wagner coexist within the brain. European Journal of Neuroscience. 2012;35(7):1190–1200. 10.1111/j.1460-9568.2011.07986.x 22487047 PMC3325511

[75] Matsumoto M, Hikosaka O. Two types of dopamine neuron distinctly convey positive and negative motivational signals. Nature. 2009;459(7248):837 10.1038/nature08028 19448610 PMC2739096

[76] Hardy NF, Buonomano DV. Neurocomputational models of interval and pattern timing. Current Opinion in Behavioral Sciences. 2016;8:250–257. 10.1016/j.cobeha.2016.01.012. 27790629 PMC5077164

[77] Merchant H, Yarrow K. How the motor system both encodes and influences our sense of time. Current Opinion in Behavioral Sciences. 2016;8:22–27. 10.1016/j.cobeha.2016.01.006.

[78] Addyman C, French RM, Thomas E. Computational models of interval timing. Current Opinion in Behavioral Sciences. 2016;8:140–146. 10.1016/j.cobeha.2016.01.004.

[79] Eagleman DM. Time and the Brain: How Subjective Time Relates to Neural Time. Journal of Neuroscience. 2005;25(45):10369–10371. 10.1523/JNEUROSCI.3487-05.2005 16280574 PMC6725822

[80] Matell MS, Meck WH, Nicolelis MAL. Interval timing and the encoding of signal duration by ensembles of cortical and striatal neurons. Behavioral Neuroscience. 2003;117(4):760–773. 10.1037/0735-7044.117.4.760 12931961

[81] Miall C. The Storage of Time Intervals Using Oscillating Neurons. Neural Computation. 1989;1(3):359–371. 10.1162/neco.1989.1.3.359

[82] Grossberg S, Schmajuk NA. Neural dynamics of adaptive timing and temporal discrimination during associative learning. Neural Networks. 1989;2(2):79–102. 10.1016/0893-6080(89)90026-9

[83] Buhusi CV, Meck WH. What makes us tick? Functional and neural mechanisms of interval timing. Nature Reviews Neuroscience. 2005;6(10):755 10.1038/nrn1764 16163383

[84] Takahashi YK, Langdon AJ, Niv Y, Schoenbaum G. Temporal Specificity of Reward Prediction Errors Signaled by Putative Dopamine Neurons in Rat VTA Depends on Ventral Striatum. Neuron. 2016;91(1):182–193. 10.1016/j.neuron.2016.05.015. 27292535 PMC4938771

[85] Daw ND, Courville AC, Touretzky DS. Representation and timing in theories of the dopamine system. Neural computation. 2006;18(7):1637–1677. 10.1162/neco.2006.18.7.1637 16764517

[86] Cools R, Frank MJ, Gibbs SE, Miyakawa A, Jagust W, D’Esposito M. Striatal Dopamine Predicts Outcome-Specific Reversal Learning and Its Sensitivity to Dopaminergic Drug Administration. Journal of Neuroscience. 2009;29(5):1538–1543. 10.1523/JNEUROSCI.4467-08.2009 19193900 PMC2940719

[87] Evers EAT, Cools R, Clark L, van der Veen FM, Jolles J, Sahakian BJ, et al Serotonergic Modulation of Prefrontal Cortex during Negative Feedback in Probabilistic Reversal Learning. Neuropsychopharmacology. 2005;30(6):1138–1147. 10.1038/sj.npp.1300663 15689962

[88] Tsuchida A, Doll BB, Fellows LK. Beyond Reversal: A Critical Role for Human Orbitofrontal Cortex in Flexible Learning from Probabilistic Feedback. Journal of Neuroscience. 2010;30(50):16868–16875. 10.1523/JNEUROSCI.1958-10.2010 21159958 PMC6634931

[89] Bari A, Theobald DE, Caprioli D, Mar AC, Aidoo-Micah A, Dalley JW, et al Serotonin Modulates Sensitivity to Reward and Negative Feedback in a Probabilistic Reversal Learning Task in Rats. Neuropsychopharmacology. 2010;35(6):1290–1301. 10.1038/npp.2009.233 20107431 PMC3055347

[90] Rudebeck PH, Murray EA. Amygdala and Orbitofrontal Cortex Lesions Differentially Influence Choices during Object Reversal Learning. Journal of Neuroscience. 2008;28(33):8338–8343. 10.1523/JNEUROSCI.2272-08.2008 18701696 PMC2556079

[91] Remijnse PL, Nielen MMA, van Balkom AJLM, Cath DC, van Oppen P, Uylings HBM, et al Reduced Orbitofrontal-Striatal Activity on a Reversal Learning Task in Obsessive-Compulsive Disorder. Archives of General Psychiatry. 2006;63(11):1225 10.1001/archpsyc.63.11.1225 17088503

[92] Izquierdo A, Jentsch JD. Reversal learning as a measure of impulsive and compulsive behavior in addictions. Psychopharmacology. 2011;219(2):607–620. 10.1007/s00213-011-2579-7 22134477 PMC3249486

[93] Bernardoni F, Geisler D, King JA, Javadi AH, Ritschel F, Murr J, et al Altered medial frontal feedback learning signals in anorexia nervosa. Biological psychiatry. 2017 10.1016/j.biopsych.2017.07.024 29025688

[94] Waltz JA, Gold JM. Probabilistic reversal learning impairments in schizophrenia: Further evidence of orbitofrontal dysfunction. Schizophrenia Research. 2007;93(1-3):296–303. 10.1016/j.schres.2007.03.010 17482797 PMC2063592

[95] O’doherty J, Dayan P, Schultz J, Deichmann R, Friston K, Dolan RJ. Dissociable roles of ventral and dorsal striatum in instrumental conditioning. science. 2004;304(5669):452–454. 10.1126/science.1094285 15087550

[96] Prevost C, McCabe JA, Jessup RK, Bossaerts P, O’Doherty JP. Differentiable contributions of human amygdalar subregions in the computations underlying reward and avoidance learning. European Journal of Neuroscience. 2011;34(1):134–145. 10.1111/j.1460-9568.2011.07686.x 21535456

[97] Wunderlich K, Beierholm UR, Bossaerts P, O’Doherty JP. The human prefrontal cortex mediates integration of potential causes behind observed outcomes. Journal of neurophysiology. 2011;106(3):1558–1569. 10.1152/jn.01051.2010 21697443 PMC3174823

[98] Evers EA, Cools R, Clark L, Van Der Veen FM, Jolles J, Sahakian BJ, et al Serotonergic modulation of prefrontal cortex during negative feedback in probabilistic reversal learning. Neuropsychopharmacology. 2005;30(6):1138 10.1038/sj.npp.1300663 15689962

[99] Allman MJ, Meck WH. Pathophysiological distortions in time perception and timed performance. Brain. 2011;135(3):656–677. 10.1093/brain/awr210 21921020 PMC3491636

[100] Radonovich KJ, Mostofsky SH. Duration judgments in children with ADHD suggest deficient utilization of temporal information rather than general impairment in timing. Child Neuropsychology. 2004;10(3):162–172. 10.1080/09297040409609807 15590495

[101] McInerney RJ, Kerns KA. Time reproduction in children with ADHD: motivation matters. Child Neuropsychology. 2003;9(2):91–108. 10.1076/chin.9.2.91.14506 12815512

[102] Smith A, Taylor E, Warner Rogers J, Newman S, Rubia K. Evidence for a pure time perception deficit in children with ADHD. Journal of Child Psychology and Psychiatry. 2002;43(4):529–542. 10.1111/1469-7610.00043 12030598

[103] Hauser TU, Iannaccone R, Ball J, Mathys C, Brandeis D, Walitza S, et al Role of the medial prefrontal cortex in impaired decision making in juvenile attention-deficit/hyperactivity disorder. JAMA psychiatry. 2014;71(10):1165–1173. 10.1001/jamapsychiatry.2014.1093 25142296

[104] Patzelt EH, Kool W, Millner AJ, Gershman SJ. Incentives Boost Model-based Control Across a Range of Severity on Several Psychiatric Constructs. Biological Psychiatry. 2018 10.1016/j.biopsych.2018.06.018 30077331 PMC6314918

[105] Voon V, Reiter A, Sebold M, Groman S. Model-based control in dimensional psychiatry. Biological psychiatry. 2017;82(6):391–400. 10.1016/j.biopsych.2017.04.006 28599832

[106] Schwartenbeck P, FitzGerald TH, Mathys C, Dolan R, Wurst F, Kronbichler M, et al Optimal inference with suboptimal models: addiction and active Bayesian inference. Medical hypotheses. 2015;84(2):109–117. 10.1016/j.mehy.2014.12.007 25561321 PMC4312353

[107] Lucantonio F, Caprioli D, Schoenbaum G. Transition from ‘model-based’to ‘model-free’behavioral control in addiction: involvement of the orbitofrontal cortex and dorsolateral striatum. Neuropharmacology. 2014;76:407–415. 10.1016/j.neuropharm.2013.05.033 23752095 PMC3809026

[108] Chiu PH, Lohrenz TM, Montague PR. Smokers’ brains compute, but ignore, a fictive error signal in a sequential investment task. Nature neuroscience. 2008;11(4):514 10.1038/nn2067 18311134

[109] Voon V, Derbyshire K, Rück C, Irvine MA, Worbe Y, Enander J, et al Disorders of compulsivity: a common bias towards learning habits. Molecular psychiatry. 2015;20(3):345 10.1038/mp.2014.44 24840709 PMC4351889

[110] Endrass T, Koehne S, Riesel A, Kathmann N. Neural correlates of feedback processing in obsessive–compulsive disorder. Journal of abnormal psychology. 2013;122(2):387 10.1037/a0031496 23421527

[111] Powers AR, Mathys C, Corlett P. Pavlovian conditioning–induced hallucinations result from overweighting of perceptual priors. Science. 2017;357(6351):596–600. 10.1126/science.aan3458 28798131 PMC5802347

