# Supplementary material for: Predicting change: Approximate inference under explicit representation of temporal structure in changing environments
Source: PLoS Comput Biol. 2019 Jan 31;15(1):e1006707. doi: 10.1371/journal.pcbi.1006707 (PMC6372216; doi:10.1371/journal.pcbi.1006707)
Supplement: S1 Table — Subject specific values of the estimated log posterior predictive model evidence (PPLME) and the corresponding model attribution (see Model fitting and model comparison for details). (PDF) [file pcbi.1006707.s001.pdf]

	PPLME		Model attribution	
subject	DU-RW	ED-HMM	DU-RW	ED-HMM
1	-13.939	-14.027	0.62	0.37
2	-9.625	-11.197	0.88	0.12
3	-9.602	-8.630	0.37	0.63
4	-9.329	-7.464	0.19	0.81
5	-19.599	-20.110	0.72	0.28
6	-16.172	-17.123	0.80	0.20
7	-9.293	-11.265	0.92	0.08
8	-33.380	-32.290	0.34	0.66
9	-12.810	-11.591	0.32	0.68
10	-17.177	-17.270	0.63	0.37
11	-7.571	-6.998	0.47	0.53
12	-2.972	-3.992	0.81	0.19
13	-3.584	-4.115	0.73	0.27
14	-19.721	-21.190	0.87	0.13
15	-15.143	-15.458	0.68	0.32
16	-11.059	-16.252	1.00	0.00
17	-8.713	-9.040	0.68	0.32
18	-19.931	-9.893	0.00	1.00
19	-5.904	-6.444	0.73	0.27
20	-13.629	-13.323	0.53	0.47
21	-17.173	-19.345	0.93	0.07
22	-4.088	-5.219	0.82	0.17
